# Chemical Constituents of Halophyte *Suaeda glauca* and Their Anti-Adipogenic and Anti-Inflammatory Activities

**DOI:** 10.3390/md24100368

**Published:** 2026-10-20

**Authors:** Chan Hee Cho, Su Ji Min, Rajath Ramachandran, Jong-Sik Jin, Changjin Lim, Hyun Joo Shim, Kiwon Jung, Ki Sung Kang, Ki Hyun Kim

**Affiliations:** 1 School of Pharmacy, Sungkyunkwan University, Suwon 16419, Republic of Korea; whckswhcks@g.skku.edu; 2 College of Korean Medicine, Gachon University, Seongnam 13120, Republic of Korea; saturn13@naver.com (S.J.M.); rajath@gachon.ac.kr (R.R.); 3 Department of Oriental Medicine Resources, Jeonbuk National University, Iksan 54596, Republic of Korea; jongsik.jin@jbnu.ac.kr; 4 School of Pharmacy, Jeonbuk National University, Jeonju 54896, Republic of Korea; limcj@jbnu.ac.kr (C.L.); shimhj@jbnu.ac.kr (H.J.S.); 5 College of Pharmacy, Seoul National University, Seoul 08826, Republic of Korea; pharmj@snu.ac.kr

**Keywords:** *Suaeda glauca*, halophyte, phenolic compounds, 3T3-L1 cells, AGS cells, IL-8

## Abstract

Halophytes are recognized as rich sources of secondary metabolites with a wide range of biological activities. To explore the phytochemical constituents and biological activities of Korean halophytes, we investigated *Suaeda glauca*, a salt-tolerant species belonging to the family Amaranthaceae. In traditional Chinese medicine, *S. glauca* has been used to reduce fever and alleviate food retention, and it has also been consumed as a food source. In the present study, we conducted a phytochemical investigation of the aerial parts of *S. glauca*. Column chromatographic separation of the CH_2_Cl_2_-soluble fraction of its hot-water extract, followed by high-performance liquid chromatography (HPLC) purification, resulted in the isolation of six compounds (**1**–**6**). Their structures were identified as vanillin (**1**), syringaldehyde (**2**), (–)-loliolide (**3**), *N*-*trans*-feruloyltyramine (**4**), *N*-*trans*-feruloylmethoxytyramine (**5**), and (–)-syringaresinol (**6**) through spectroscopic analyses and comparison with previously reported data. Notably, this study represents the first report of compounds **1**–**3** and **6** being isolated from *S. glauca*. The isolated compounds were evaluated for their anti-adipogenic and anti-inflammatory activities using 3T3-L1 adipocytes and TNF-α-stimulated AGS cells, respectively. Compound **2** suppressed adipocyte differentiation, accompanied by reduced lipid accumulation and decreased expression of PPARγ, C/EBPα, and adiponectin, whereas compound **6** attenuated TNF-α-induced inflammatory responses by reducing IL-8 production and ERK/p38 MAPK activation. To conceptualize these biological effects, computational network pharmacology and molecular docking were employed, identifying PTGS2 (COX-2) as a theoretical mechanistic bridge linking the PPARγ metabolic axis and the MAPK inflammatory cascade. Collectively, these findings expand the phytochemical knowledge of *S. glauca* and provide structurally supported hypothesis for the dual-axis modulation of metaflammation by its major active constituents.

## 1. Introduction

Obesity is a chronic metabolic disorder characterized by excessive fat accumulation and is closely associated with various metabolic diseases, including type 2 diabetes, dyslipidemia, cardiovascular disease, and non-alcoholic fatty liver disease [1]. Adipogenesis, the process by which preadipocytes differentiate into mature adipocytes, plays a central role in the development of obesity. Accordingly, the regulation of adipocyte differentiation has emerged as an important strategy for the discovery of novel anti-obesity agents [2,3].

Gastric inflammation is another major health concern and is closely associated with the development of chronic gastritis and other gastroduodenal disorders. Gastric epithelial cells are not merely a physical barrier but actively participate in mucosal immune responses by sensing inflammatory stimuli and producing cytokines and chemokines that regulate local inflammation [4]. AGS cells, a human gastric adenocarcinoma-derived epithelial cell line, are widely used as an in vitro model to investigate inflammatory responses in gastric epithelial cells. In particular, TNF-α, a major pro-inflammatory cytokine, can activate inflammatory signaling pathways, including MAPK and NF-κB, and stimulate the production of chemokines such as IL-8 in gastric epithelial cells. IL-8 plays an important role in neutrophil recruitment and activation, thereby contributing to gastric mucosal inflammation [5,6]. Accordingly, TNF-α-stimulated AGS cells provide a useful experimental model for evaluating cytokine-mediated inflammatory responses, with IL-8 secretion serving as a measurable indicator of inflammatory activation. Based on this rationale, the present study employed TNF-α-stimulated AGS cells to evaluate the anti-inflammatory activities of the isolated compounds by measuring IL-8 production. Natural products capable of modulating inflammatory signaling pathways have attracted considerable attention as potential therapeutic agents for gastric inflammatory diseases [7,8].

Despite advances in pharmacological interventions, obesity and gastric inflammatory disorders remain challenging to manage because of their multifactorial pathogenesis and the limitations associated with long-term treatment. Natural products continue to represent an important source of structurally diverse bioactive compounds for the development of functional foods and therapeutic agents [9,10,11]. In particular, halophytes, which thrive under extreme saline environments, are known to produce a wide range of secondary metabolites that contribute to stress adaptation and exhibit diverse biological activities [12,13,14]. Therefore, halophytic plants have emerged as promising resources for the discovery of natural products with anti-adipogenic and anti-inflammatory potential.

To explore the phytochemical diversity of Korean halophytes, several halophytic species were collected, and *Suaeda glauca* was selected for detailed phytochemical investigation because of its reported medicinal use and pharmacological potential. *S. glauca*, an obligate halophyte belonging to the family Amaranthaceae (previously classified within Chenopodiaceae), is widely distributed in seawater-influenced environments, including tidal flats, salt marshes, and coastal saline–alkaline lands across East Asia [15]. In Korea, *S. glauca* occurs in coastal salt marshes and tidal-flat habitats along the western and southern coasts, where plant communities are influenced by soil salinity and periodic tidal inundation [16,17]. As a coastal halophyte, *S. glauca* possesses physiological and molecular adaptations that enable it to tolerate high-salinity environments, including ion homeostasis and osmotic regulation [18]. Owing to its coastal habitat, *S. glauca* has been recorded among the marine medicinal resources of traditional Chinese medicine, in which it has long been used to reduce fever and alleviate food retention, and it has also been consumed as a food source [19]. Previous studies have reported that *S. glauca* exhibits various biological activities, including antioxidant, anti-fibrotic, and hepatoprotective effects [20,21,22]. Its flavonoid constituents have also been reported to regulate enzymes associated with hair loss [23]. Phytochemical investigations have identified several classes of secondary metabolites from *S. glauca*, including phenolic compounds, flavonoids, terpenoids, and alkaloids [24]. Furthermore, GC–MS-based comparative metabolomic profiling has revealed differences in the accumulation of sugars, amino acids, organic acids, and other metabolites associated with saline–alkaline stress adaptation in *S. glauca* [25]. In plants, salt-induced oxidative stress can influence the phenylpropanoid pathway and the accumulation of phenolic metabolites involved in antioxidant defense and stress adaptation [12,26]. Consistent with the importance of secondary metabolism in stress responses, genomic analyses of *S. glauca* have provided insights into salt-responsive molecular pathways, including those associated with flavonoid and lignin biosynthesis [18]. However, these phytochemical studies have primarily focused on extracts prepared using organic solvents, whereas the chemical constituents of hot-water extracts remain relatively less characterized. Moreover, the biological roles and molecular pathways associated with individual constituents of *S. glauca* have not been sufficiently characterized. In particular, limited information is available regarding their effects on adipocyte differentiation in 3T3-L1 cells and on inflammatory signaling in gastric epithelial cells. Therefore, investigation of the hot-water extract and its bioactive constituents may broaden the phytochemical profile of *S. glauca* and provide mechanistic insight into their biological activities.

To address this gap, we conducted a phytochemical investigation of the aerial parts of *S. glauca*. Column chromatographic separation of the CH_2_Cl_2_-soluble fraction of the hot-water extract, followed by high-performance liquid chromatography (HPLC) purification, resulted in the isolation of six compounds (**1**–**6**). Their structures were identified by comprehensive spectroscopic analyses, including NMR spectroscopy and high-resolution electrospray ionization mass spectrometry (HR-ESI-MS). The isolated compounds (**1**–**6**) were subsequently evaluated for their effects on adipocyte differentiation in 3T3-L1 cells and TNF-α-induced inflammatory responses in AGS cells. For the most active compounds, key adipogenic regulators and inflammation-related signaling pathways were further examined to define the pathway-level molecular events associated with their biological activities.

## 2. Results

### 2.1. Isolation and Structural Elucidation of Compounds (**1**–**6**)

**Figure 1:**
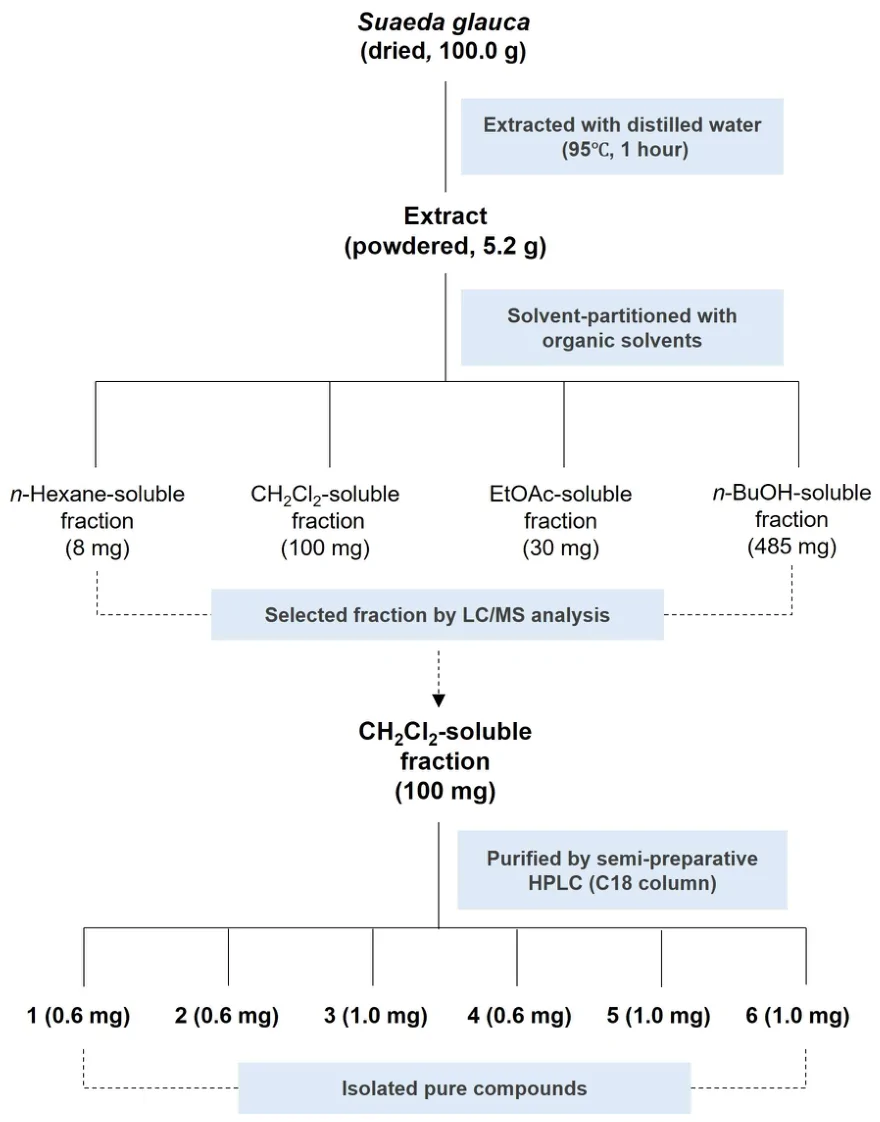
Isolation scheme of compounds **1**–**6** from the CH_2_Cl_2_-soluble fraction of the hot-water extract of *S. glauca*.

**Figure 2:**
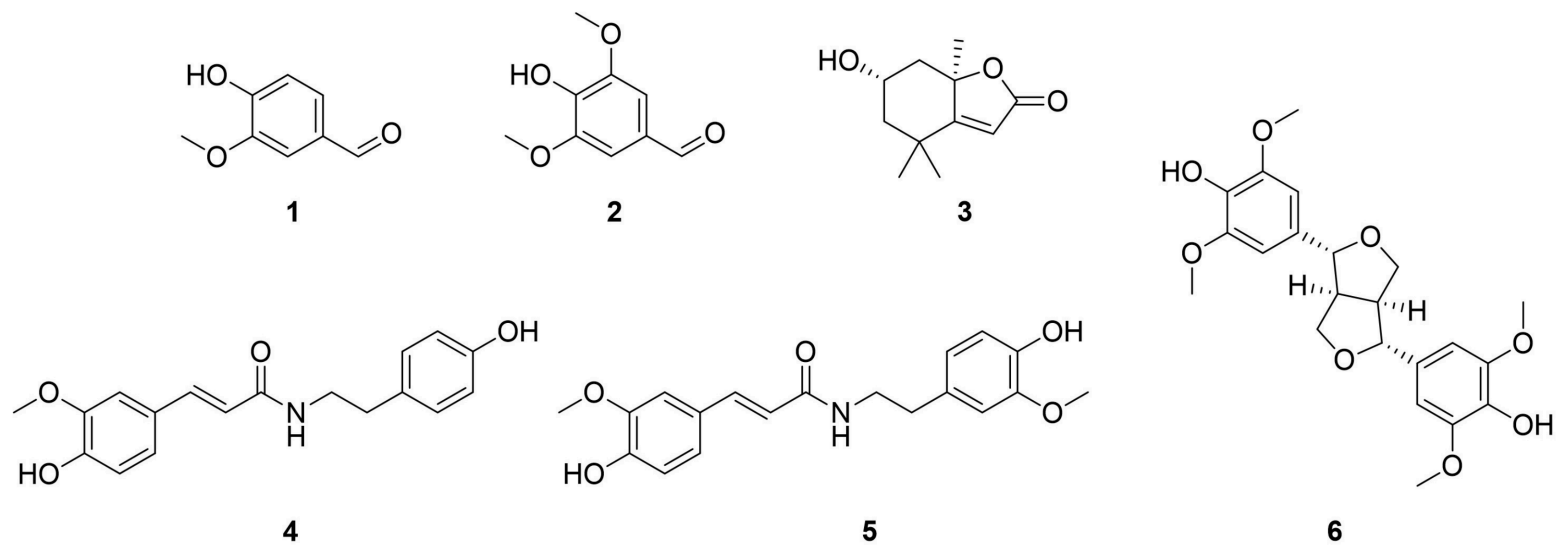
The chemical structures of compounds **1**–**6**.

The crude extract of *S. glauca* was suspended in distilled water and successively partitioned with four organic solvents of increasing polarity, namely *n*-hexane, CH_2_Cl_2_, EtOAc, and *n*-BuOH, yielding four corresponding fractions (Figure 1). LC–MS analysis of these fractions, supported by a custom-built UV spectral library [27,28], indicated that the CH_2_Cl_2_-soluble fraction was enriched in major phenolic constituents. Phenolic compounds are well known for their diverse pharmacological and nutritional properties, including antimicrobial, antibacterial, antiviral, anti-atherosclerotic, antioxidant, and anti-inflammatory activities, and have therefore attracted considerable scientific interest [29,30,31,32]. Accordingly, the CH_2_Cl_2_-soluble fraction was selected for further purification. Semi-preparative reversed-phase HPLC separation of the CH_2_Cl_2_ fraction led to the isolation of six compounds (**1**–**6**) (Figure 1). Their structures were elucidated based on NMR spectroscopic and HR-ESI-MS data, together with comparison with previously reported values, and were identified as vanillin (**1**) [33], syringaldehyde (**2**) [34], (–)-loliolide (**3**) [35], *N*-*trans*-feruloyltyramine (**4**) [36], *N*-*trans*-feruloylmethoxytyramine (**5**) [36], and (–)-syringaresinol (**6**) [37] (Figure 2). To the best of our knowledge, compounds **1**–**3** and **6** are reported herein from *S. glauca* for the first time.

### 2.2. Anti-Adipogenic Effects of Isolated Compounds **1**–**6** in 3T3-L1 Cells

**Figure 3:**
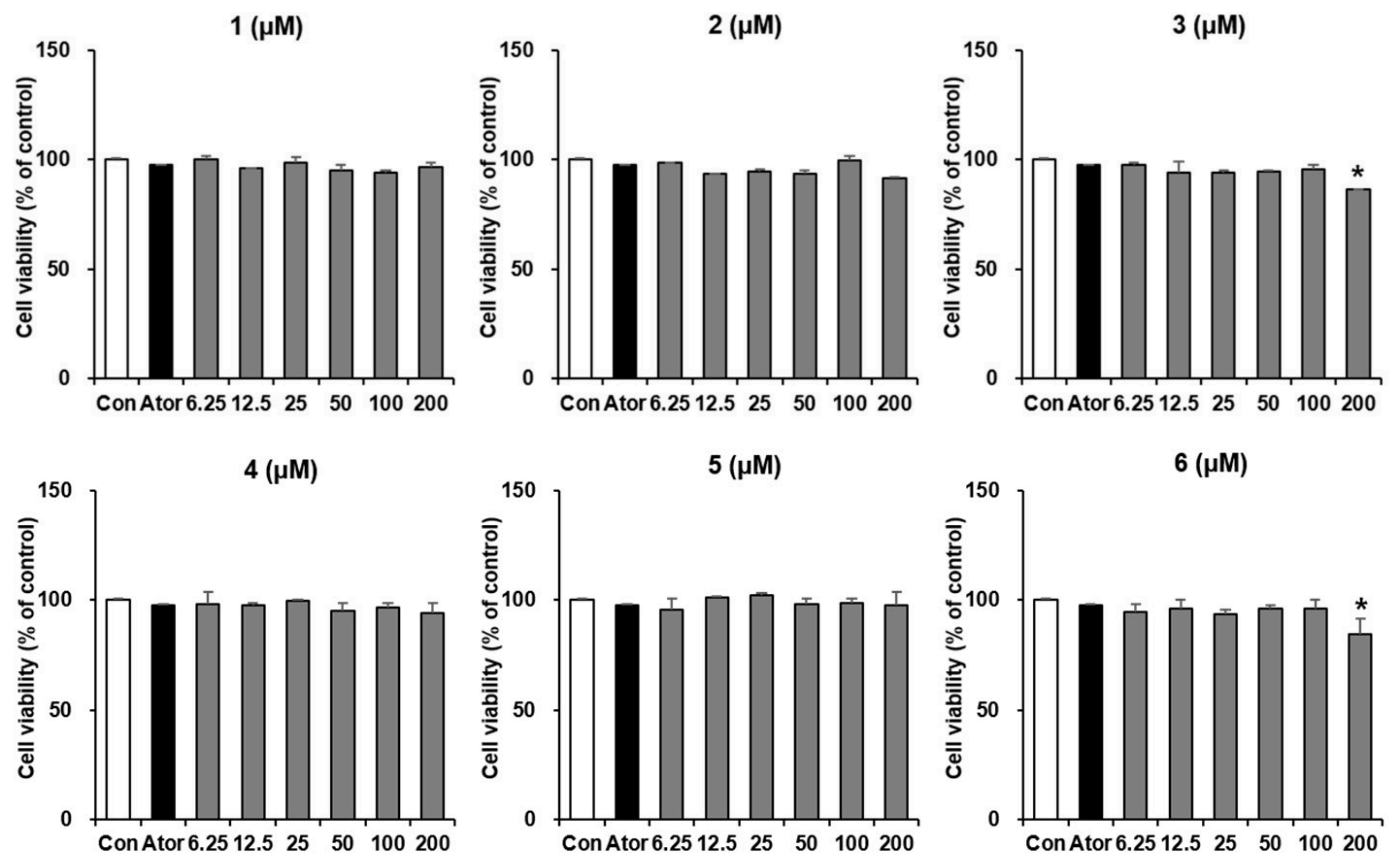
Effects of compounds **1**–**6** on the viability of 3T3-L1 cells. 3T3-L1 cells were treated with compounds **1**–**6** at concentrations ranging from 6.25 to 200 μM for 24 h. Cell viability was assessed using the EZ-Cytox assay and expressed as a percentage of the untreated control group. Data are presented as the mean ± SD (*n* = 3). Statistical significance was determined relative to the untreated control group (* *p* < 0.05). Ator indicates atorvastatin, which was used as a positive control.

The effects of compounds **1**–**6** on the viability of 3T3-L1 preadipocytes were evaluated to determine appropriate non-cytotoxic concentrations for subsequent adipogenesis assays. Compounds **1**, **2**, **4**, and **5** did not significantly affect cell viability at concentrations up to 200 μM, whereas compounds **3** and **6** showed significant cytotoxicity at 200 μM (Figure 3). Accordingly, non-cytotoxic concentrations of each compound were selected for the subsequent experiments. These results provide a basis for interpreting the subsequent changes in adipogenic responses as compound-related biological effects rather than consequences of nonspecific cytotoxicity or reduced cell number.

**Figure 4:**
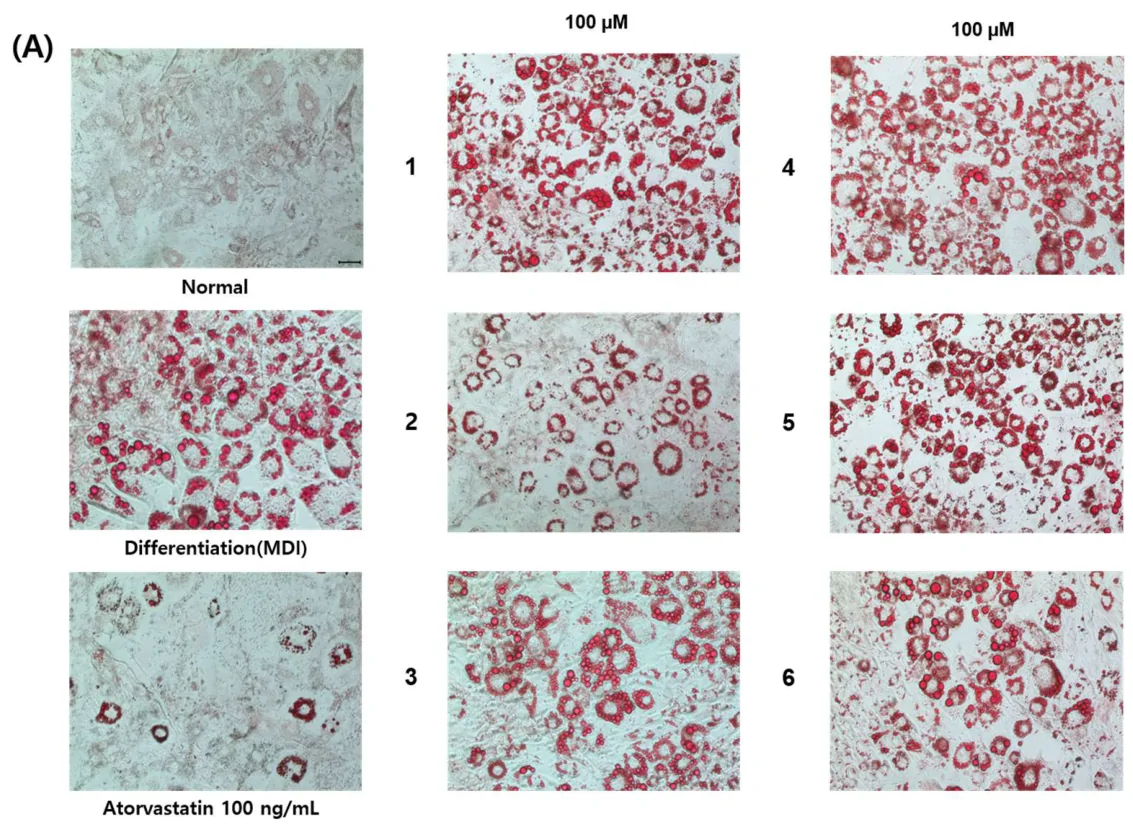

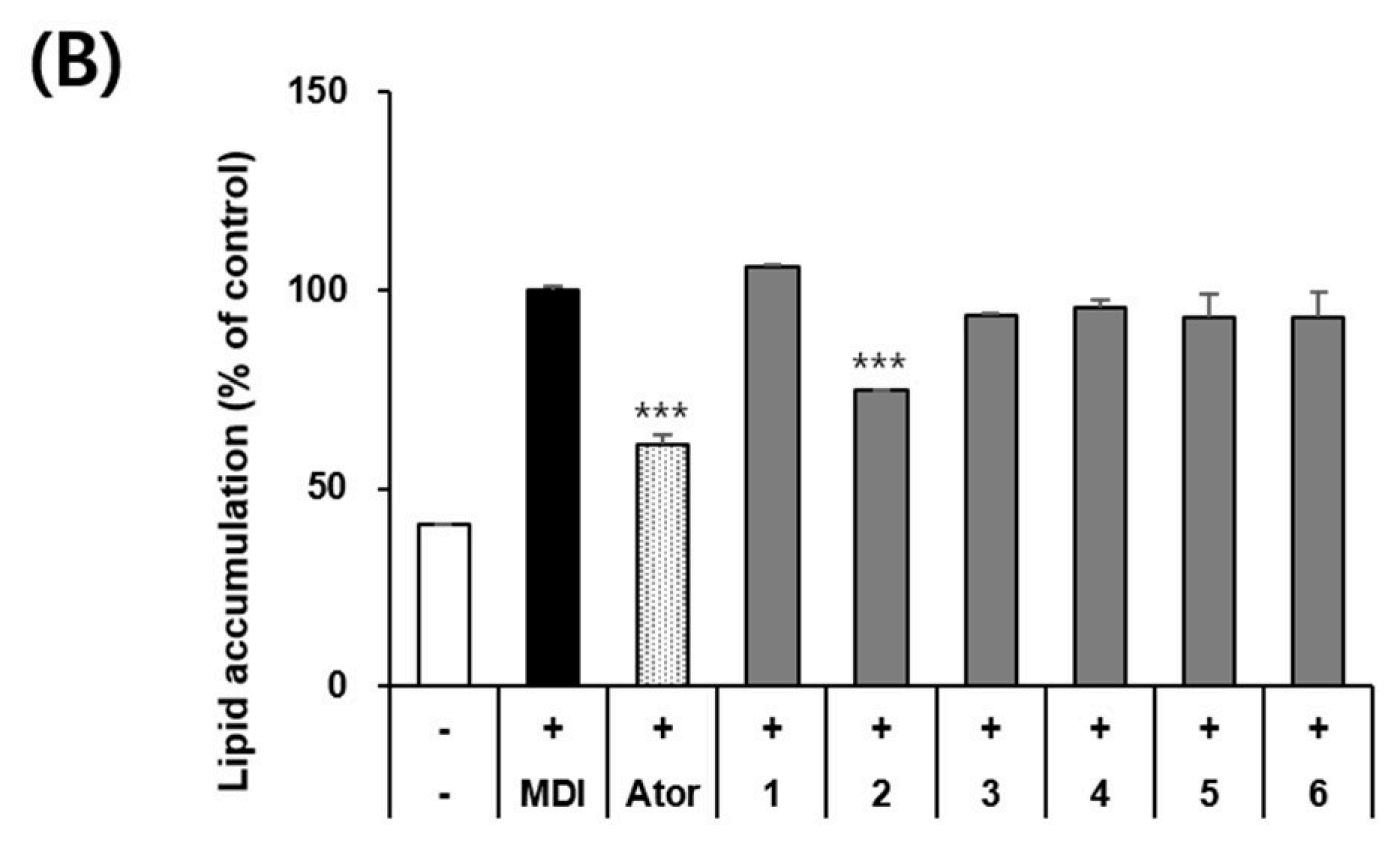
Effects of compounds **1**–**6** on lipid accumulation in 3T3-L1 adipocytes. 3T3-L1 preadipocytes were treated with compounds **1**–**6** (100 μM) throughout the differentiation period, and intracellular lipid accumulation was evaluated on day 8 using Oil Red O staining. (**A**) Representative images of Oil Red O-stained adipocytes. (**B**) Quantitative analysis of intracellular lipid accumulation. Data are presented as the mean ± SD (*n* = 3). *** *p* < 0.001 versus the MDI-induced differentiated control group. (−), non-differentiated control; (+), MDI-induced differentiated control; Ator, atorvastatin (positive control); **1**–**6**, compound-treated groups.

The effects of compounds **1**–**6** on lipid accumulation during adipocyte differentiation were evaluated in 3T3-L1 cells using Oil Red O staining. As shown in Figure 4, treatment with compounds **1**–**6** at 100 μM revealed that compound **2** significantly reduced intracellular lipid accumulation compared with the differentiated control group. In contrast, the other compounds exhibited little or no inhibitory effect on lipid accumulation. These results identify compound **2** as the most active compound in reducing lipid accumulation under the present experimental conditions.

**Figure 5:**
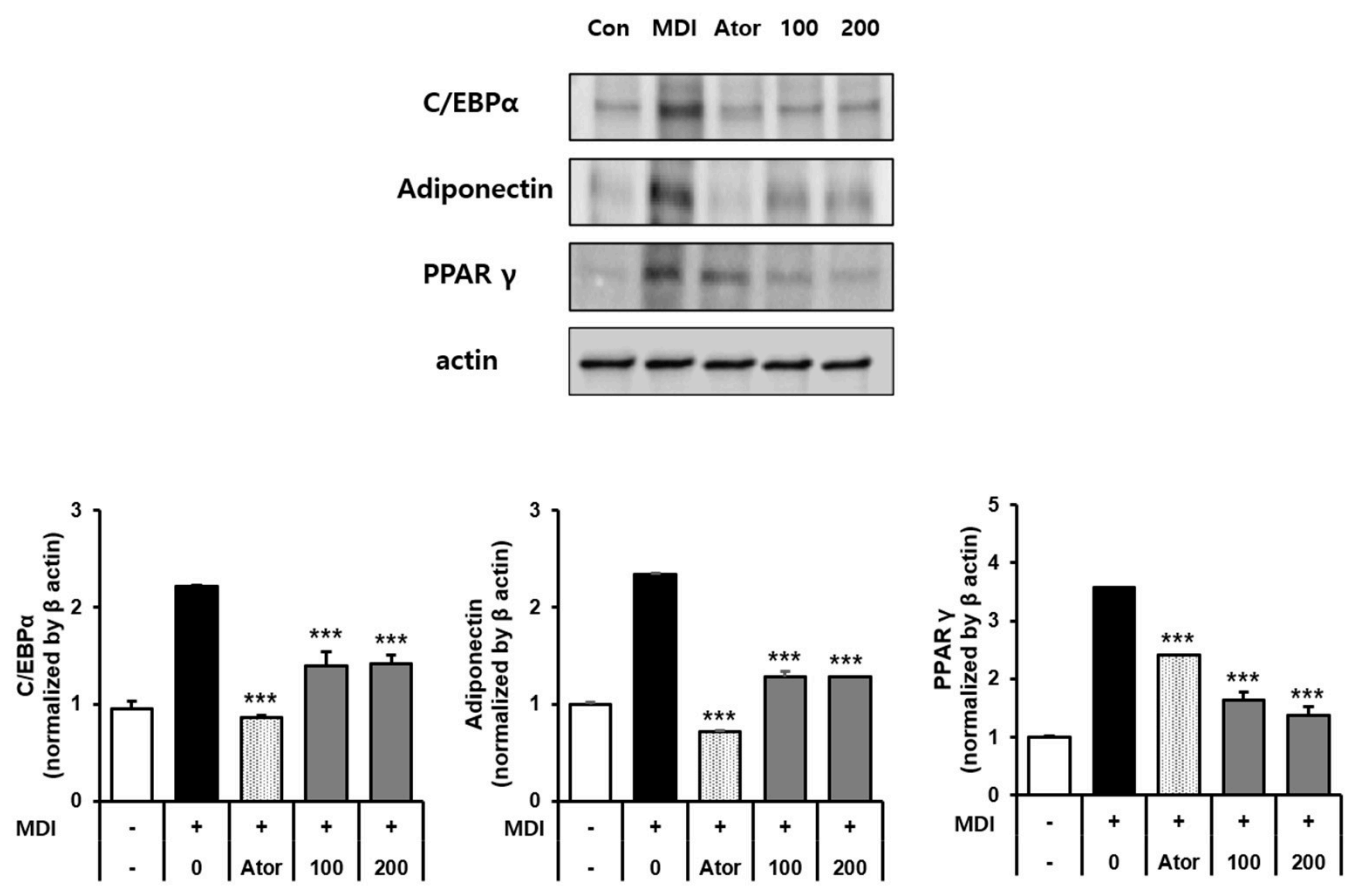
Effects of compound **2** on the expression of adipogenic proteins in 3T3-L1 adipocytes. 3T3-L1 preadipocytes were treated with compound **2** (100 and 200 μM) or atorvastatin (100 ng/mL) throughout the differentiation period. On day 8, the expression levels of adipogenic proteins were analyzed by Western blotting. Data are presented as the mean ± SD (*n* = 3). *** *p* < 0.001 versus the MDI-induced differentiated control group. (−), non-differentiated control; (+), MDI-induced differentiated control; MDI, differentiation medium containing dexamethasone, 3-isobutyl-1-methylxanthine (IBMX), and insulin; Ator, atorvastatin. 100 and 200 indicate treatment with compound **2** at 100 and 200 μM, respectively.

Next, the effects of compound **2** on the expression of adipogenesis-related proteins were investigated in differentiated 3T3-L1 adipocytes by Western blot analysis. As shown in Figure 5, treatment with compound **2** (100 and 200 μM) decreased the expression levels of peroxisome proliferator-activated receptor gamma (PPARγ), CCAAT/enhancer-binding protein alpha (C/EBPα), and adiponectin in a concentration-dependent manner. Atorvastatin, used as a positive control, also reduced the expression of these adipogenic markers. These results demonstrate that compound 2 downregulated key adipogenic regulators, PPARγ and C/EBPα, together with the mature adipocyte marker adiponectin.

### 2.3. Anti-Inflammatory Effects of Isolated Compounds **1**–**6** in TNF-α-Stimulated AGS Cells

**Figure 6:**
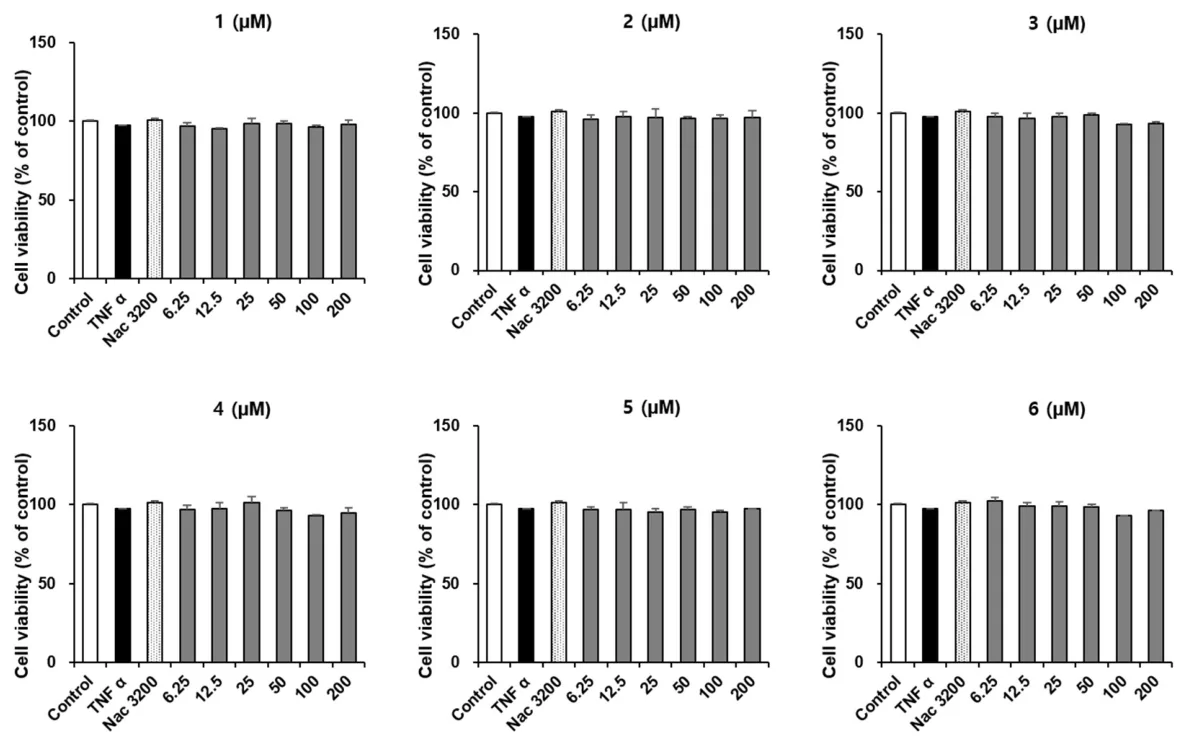
Effects of isolated compounds **1**–**6** on the viability of AGS cells. AGS cells were treated with compounds **1**–**6** at the indicated concentrations for 24 h. Cell viability was assessed using the EZ-Cytox assay and expressed as a percentage of the untreated control group. Data are presented as the mean ± SD (*n* = 3). No significant differences were observed compared with the untreated control group. TNF-α, tumor necrosis factor-α (20 ng/mL); Nac, *N*-acetyl-L-cysteine (3200 μg/mL, positive control).

Given the well-documented anti-inflammatory properties of phenolic compounds [28,29], the isolated compounds were further evaluated for their effects on TNF-α-induced inflammatory responses in AGS gastric epithelial cells. Prior to assessing their anti-inflammatory activities, the effects of compounds **1**–**6** on AGS cell viability were examined to determine appropriate concentrations for subsequent experiments. As shown in Figure 6, treatment with compounds **1**–**6** at the tested concentrations did not significantly affect cell viability compared with the untreated control group. These findings indicate that the selected concentrations were non-cytotoxic and provide a basis for interpreting subsequent changes in IL-8 production and inflammatory signaling as compound-related biological effects rather than consequences of nonspecific cytotoxicity or reduced cell number.

**Figure 7:**
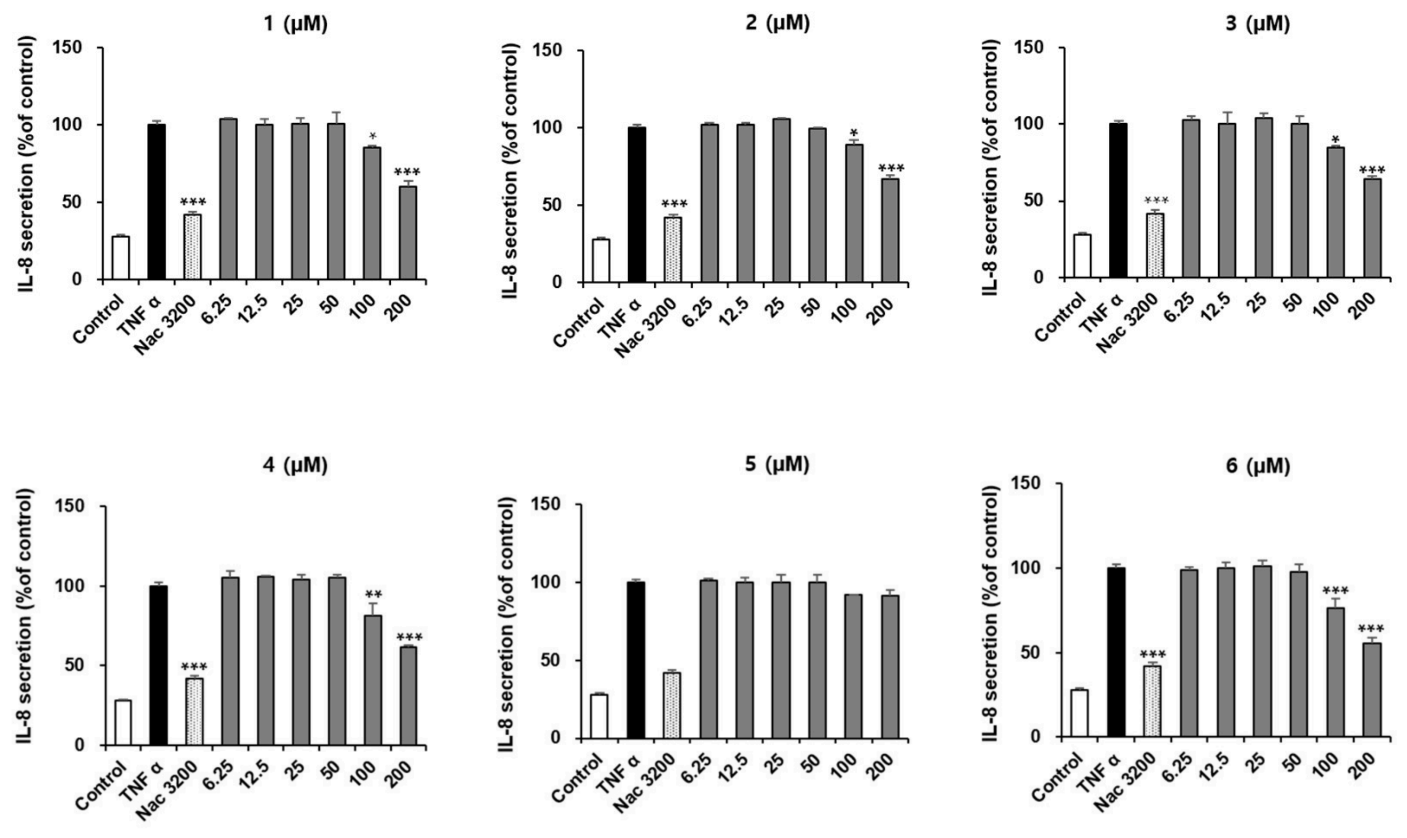
Effects of compounds **1**–**6** on TNF-α-induced IL-8 secretion in AGS cells. AGS cells were pretreated with compounds **1**–**6** (6.25–200 μM) or N-acetyl-L-cysteine (NAC, 3200 μg/mL) and subsequently stimulated with TNF-α (20 ng/mL). The levels of IL-8 released into the culture medium were quantified using an ELISA kit. Data are presented as the mean ± SD (*n* = 3). Statistical significance was determined relative to the TNF-α-stimulated control group (* *p* < 0.05, ** *p* < 0.01, and *** *p* < 0.001).

The effects of compounds **1**–**6** on TNF-α-induced IL-8 secretion were evaluated in AGS cells. As shown in Figure 7, stimulation with TNF-α significantly increased IL-8 secretion compared with the untreated control group. In contrast, N-acetyl-L-cysteine (NAC, 3200 μg/mL), used as a positive control, significantly suppressed TNF-α-induced IL-8 secretion. Treatment with compounds **1**–**6** at 100 and 200 μM reduced IL-8 production to varying extents, with the exception of compound **5**. Among the tested compounds, compound **6** exhibited the most potent inhibitory effect on IL-8 secretion and was therefore selected for further investigation of the signaling pathways associated with its anti-inflammatory activity.

**Figure 8:**
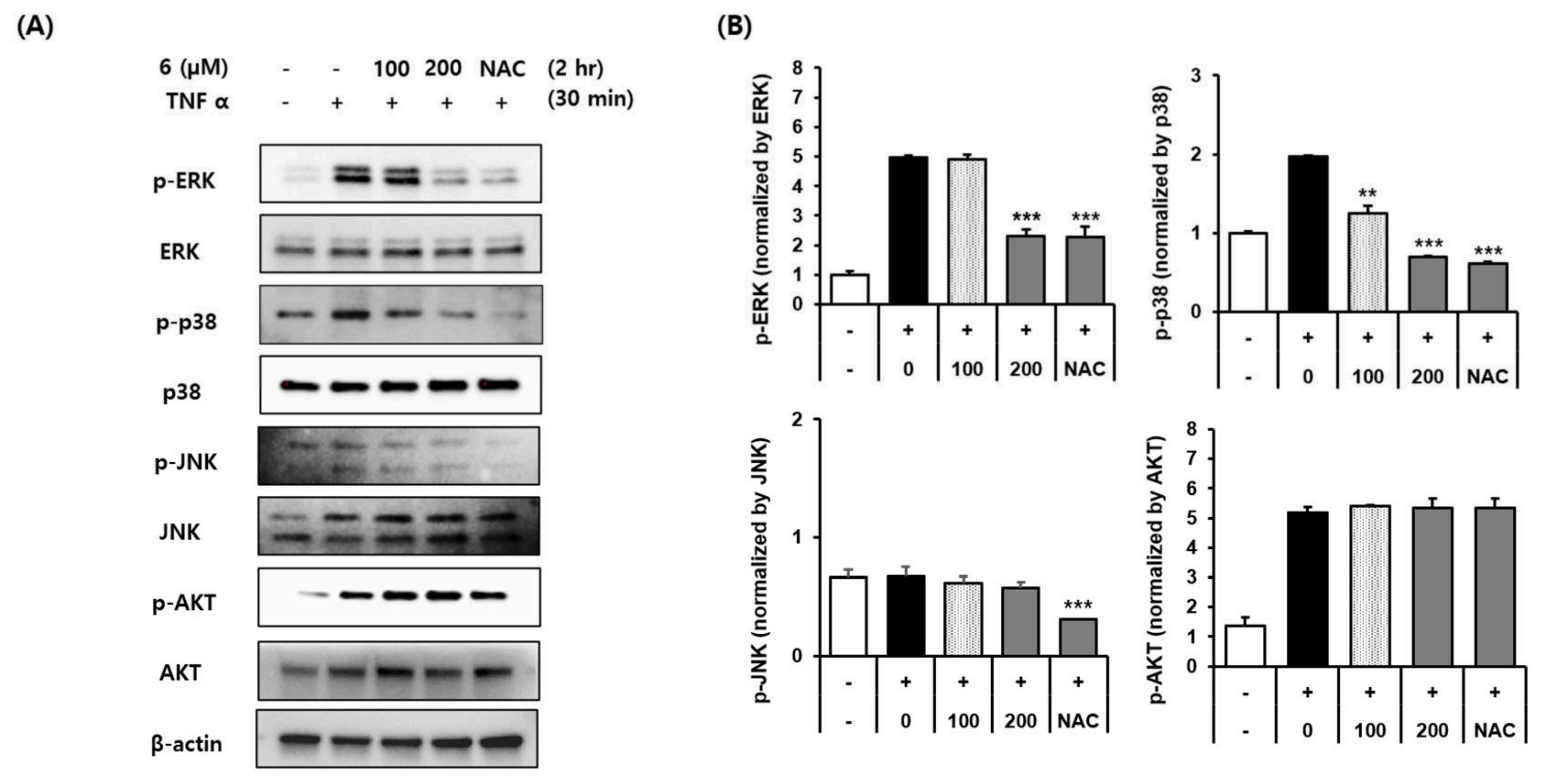
Effects of compound **6** on TNF-α-induced MAPK and AKT phosphorylation in AGS cells. AGS cells were pretreated with compound **6** (100 or 200 μM) or N-acetyl-L-cysteine (NAC, 3200 μg/mL, positive control) and subsequently stimulated with TNF-α (20 ng/mL). (**A**) Representative Western blot images of phosphorylated and total ERK, p38, JNK, and AKT. (**B**) Densitometric analysis of phosphorylated protein levels normalized to the corresponding total protein levels. Data are presented as the mean ± SD (*n* = 3). ** *p* < 0.01, and *** *p* < 0.001 versus the TNF-α-stimulated control group. 100 and 200 indicate treatment with compound **6** at 100 and 200 μM, respectively.

Next, the effects of compound **6** on TNF-α-induced MAPK and AKT signaling pathways were investigated in AGS cells by Western blot analysis. As shown in Figure 8, TNF-α stimulation markedly increased the phosphorylation levels of ERK, p38, JNK, and AKT compared with the untreated control group. In contrast, N-acetyl-L-cysteine (NAC, 3200 μg/mL), used as a positive control, reduced the phosphorylation of these signaling proteins. Treatment with compound **6** significantly suppressed TNF-α-induced phosphorylation of ERK and p38, whereas no appreciable effects were observed on JNK or AKT phosphorylation. The total protein levels of ERK, p38, JNK, and AKT remained unchanged among the experimental groups. These findings suggest that compound **6** attenuates TNF-α-induced inflammatory signaling in AGS cells, at least in part, through the inhibition of ERK- and p38-mediated signaling pathways.

### 2.4. Target Identification and Gene Intersection

The molecular framework for distinct metabolic and inflammatory activities observed in vitro was investigated via network pharmacology. By cross-referencing the DisGeNET datasets for obesity and gastric inflammation, we identified 48 shared disease-associated genes (Supplementary File S1). SwissTargetPrediction further identified 40 predicted macromolecular targets shared by compounds **2** and **6**. Integration of the predicted compound targets with the disease-associated gene set identified *PTGS2* (COX-2) as a common candidate target. Given the established involvement of *PTGS2* in inflammatory and metabolic processes, this predictive intersection suggests a potential molecular link between the independent biological effects of compound **2** (anti-adipogenic) and compound **6** (anti-inflammatory) examined in this study. However, the involvement of PTGS2 remains predictive and requires experimental validation.

### 2.5. Molecular Docking of Compounds **2** and **6** with Selected Protein Targets

**Figure 9:**
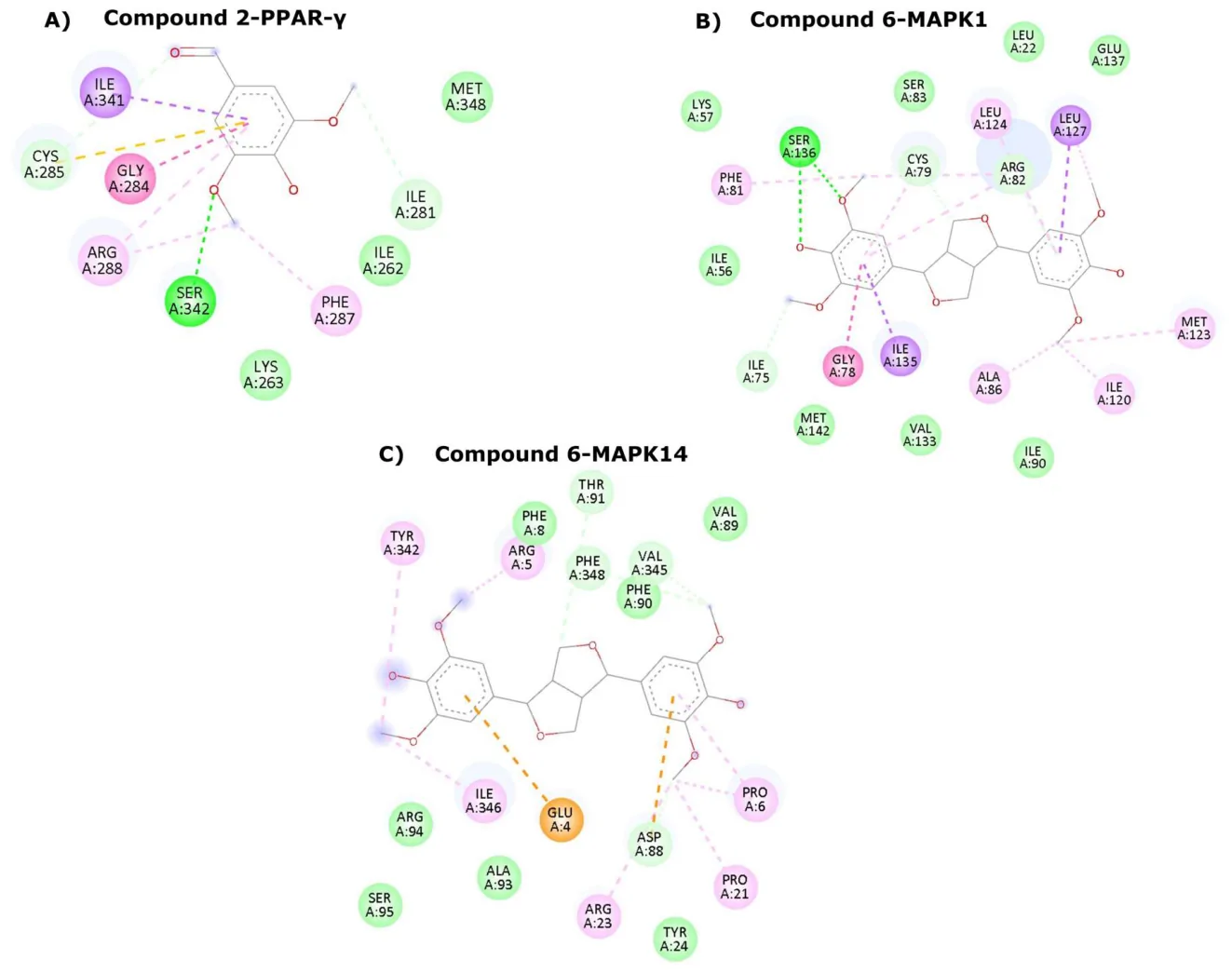
(**A**) Binding pose of compound **2** in the active site of PPAR-γ. The ligand is anchored by a conventional hydrogen bond with Ser342, with the aromatic core stabilized by a network of π interactions. (**B**) Binding pose of compound **6** at the active site of MAPK1. The interaction is driven by a critical hydrogen bond at Ser136 and extensive hydrophobic packing (π-alkyl/alkyl) with surrounding residues. (**C**) Binding pose of compound **6** in the active site of MAPK14. The complex is stabilized by distinct hydrogen bonding rigidly supported by π-anion contacts at Glu4 and Asp88, and multiple π-alkyl interactions. Different color codes indicate various interactions and as follows: Bright green line-conventional hydrogen bond, purple line—π-Alkyl/π-Sigma, yellow line—π-Sulphur, pink line—π-Alkyl/Hydrophobic, orange line—electrostatic interaction, light green (without lines)—Van der Waals.

Computational docking was conducted to explore potential interactions between the biologically active compounds (**2** and **6**) and selected proteins associated with the experimentally observed biological effects, including PPARγ, MAPK1 (ERK2), and MAPK14 (p38α). The predicted docking scores and molecular interactions are summarized in Table 1. Compound **2** exhibited a docking score of −5.306 kcal/mol within the ligand-binding pocket of PPARγ. The predicted binding pose involved a hydrogen bond with Ser342 and several hydrophobic and π-mediated interactions with surrounding residues, including Gly284, Arg288, Phe287, and Ile341. Additional interactions involving Cys285, Met348, Ile281, Ile262, and Lys263 were also predicted to contribute to the stabilization of the ligand within the binding pocket (Figure 9A). Although these findings suggest that compound **2** can be accommodated within the PPARγ ligand-binding pocket, the moderate docking score does not provide sufficient evidence of direct binding or functional modulation of PPARγ. Further biochemical binding and target-engagement studies are required to validate this potential interaction and clarify its relevance to the observed anti-adipogenic activity.

Compound **6** exhibited a predicted docking score of −8.117 kcal/mol with MAPK1. The predicted binding pose involved a hydrogen bond with Ser136 and multiple hydrophobic interactions with surrounding residues, including Cys79, Arg82, Leu124, Leu127, Gly78, Ile135, Ala86, Met123, Ile120, and Phe81 (Figure 9B). Similarly, compound **6** exhibited a docking score of −7.206 kcal/mol with MAPK14, with predicted hydrogen-bonding interactions involving Phe90, Thr91, and Phe348, along with additional electrostatic and hydrophobic interactions involving surrounding residues (Figure 9C). These docking results suggest potential interactions between compound **6** and MAPK1/MAPK14, providing preliminary structural hypotheses that may be relevant to the experimentally observed attenuation of ERK and p38 phosphorylation. However, these computational predictions do not establish direct target binding or kinase inhibition, and further experimental validation is required to clarify the molecular mechanisms underlying the observed anti-inflammatory effects.

## 3. Discussion

The present study expands the phytochemical profile of *S. glauca*, a coastal halophyte that has been extensively investigated for its physiological adaptation to saline and alkaline stress [38,39]. The identification of six compounds, including four constituents reported from this species for the first time, further highlights the chemical diversity of *S. glauca* and its potential as a source of biologically active natural products. Notably, the biological evaluation identified syringaldehyde (**2**) and (−)-syringaresinol (**6**) as the most active constituents in the anti-adipogenic and anti-inflammatory screening assays, respectively, providing a basis for further investigation of their associated molecular pathways. These findings suggest that distinct constituents of *S. glauca* may influence different cellular processes associated with metabolic regulation and inflammatory responses. To further explore potential molecular relationships between these biological activities, network-based target prediction was integrated with the experimental findings. This analysis identified *PTGS2* (COX-2) as a potential common candidate target associated with obesity and gastric inflammation. Given the established involvement of COX-2 in inflammatory responses and metabolic dysfunction, this prediction suggests a possible molecular connection between the biological processes investigated in this study. However, the involvement of PTGS2 remains hypothetical, as its direct modulation by compounds **2** and **6** was not experimentally evaluated. Thus, the integration of cellular and computational findings provides a preliminary framework for understanding the potential biological significance of these constituents, while further experimental studies are required to establish their precise molecular targets and mechanisms of action.

The anti-adipogenic activity of the isolated compounds was evaluated in 3T3-L1 cells by assessing lipid accumulation during adipocyte differentiation. Among compounds **1**–**6**, compound **2** exhibited the greatest reduction in lipid accumulation, decreasing it to 74.80% of the MDI-treated control (a 25.20% reduction), whereas the corresponding values for the other compounds ranged from 92.77% to 105.83%. This quantitative difference provided the basis for selecting compound **2** for further investigation of adipogenesis-related molecular markers. Subsequent Western blot analysis revealed that compound **2** reduced the expression of PPARγ, C/EBPα, and adiponectin. PPARγ and C/EBPα are key transcriptional regulators that cooperatively control adipocyte differentiation and adipocyte-specific gene expression, whereas adiponectin is a characteristic adipokine of mature adipocytes that contributes to metabolic homeostasis [3,40,41]. Although adiponectin is generally recognized for its insulin-sensitizing and anti-inflammatory properties, its reduced expression in the present differentiation model should be interpreted in the context of the concurrent decreases in PPARγ and C/EBPα expression and intracellular lipid accumulation. These coordinated changes suggest that compound **2** suppresses the adipogenic differentiation program and subsequent adipocyte maturation, rather than merely reducing cellular lipid content. Importantly, the reduction in adiponectin expression should not itself be interpreted as a beneficial metabolic effect, as its implications for insulin sensitivity and mature adipocyte function remain to be determined.

Compound **2** was identified as syringaldehyde, a phenolic aldehyde whose previously reported biological activities have predominantly focused on antioxidant, anti-inflammatory, and metabolic effects. However, its role in the molecular regulation of adipocyte differentiation remains less well characterized. Thus, the present findings extend the biological characterization of syringaldehyde by associating its anti-adipogenic activity with attenuation of the PPARγ/C/EBPα-regulated adipogenic differentiation program. To further explore potential molecular interactions, docking analysis was performed using PPARγ as a candidate protein target. Compound **2** was predicted to fit within the PPARγ ligand-binding pocket, with a docking score of −5.306 kcal/mol and a predicted hydrogen-bonding interaction involving Ser342. Although this result provides a preliminary structural hypothesis for a possible interaction with PPARγ, it does not establish direct binding or functional modulation of the receptor. The observed reduction in PPARγ expression may reflect suppression of the adipogenic differentiation program rather than direct inhibition of PPARγ itself**.** Further biochemical binding and target-engagement studies are therefore required to determine whether PPARγ is a direct molecular target of compound **2** and to clarify its contribution to the observed anti-adipogenic activity.

To investigate the anti-inflammatory effects of the isolated compounds in gastric epithelial cells, TNF-α-stimulated AGS cells were employed as an in vitro inflammatory model. IL-8 is a major pro-inflammatory chemokine involved in gastric mucosal inflammation through the recruitment and activation of inflammatory cells, making its secretion a useful indicator of inflammatory responses in gastric epithelial cells [8,42]. Among the six isolated compounds, compound **6** exhibited the greatest numerical inhibition of TNF-α-induced IL-8 secretion (44.26% at 200 μM), compared with inhibition rates ranging from 8.49% to 39.95% for the other compounds. This quantitative comparison provided the basis for selecting compound **6** for further investigation of inflammation-related signaling pathways.

To explore the molecular events associated with its anti-inflammatory activity, the effects of compound **6** on TNF-α-induced MAPK and AKT signaling were examined. Compound **6** significantly reduced ERK and p38 phosphorylation without markedly affecting JNK or AKT phosphorylation, while the total protein levels remained unchanged. ERK and p38 are key components of MAPK signaling involved in inflammatory responses and the regulation of IL-8 production in gastric epithelial cells [6,43,44]. In contrast, AKT participates in the PI3K/AKT signaling pathway, which regulates various cellular processes, including survival and inflammation-related responses [45]. The concurrent reduction in ERK and p38 phosphorylation without changes in their total protein levels suggests that compound **6** attenuates the activation of these signaling proteins rather than altering their overall expression. Furthermore, the absence of marked changes in JNK and AKT phosphorylation suggests that compound **6** does not broadly suppress all the signaling pathways examined but preferentially attenuates ERK/p38-associated signaling under the present experimental conditions. Together with the observed reduction in IL-8 secretion, these findings suggest that the anti-inflammatory activity of compound **6** is associated, at least in part, with attenuation of ERK/p38-related inflammatory signaling. However, further pathway-specific studies would be required to establish whether the modulation of ERK and p38 directly mediates the inhibition of IL-8 production.

Compound **6** was identified as (−)-syringaresinol, a furofuran-type lignan with previously reported antioxidant, anti-inflammatory, and cytoprotective activities. Previous research demonstrated that (+)-syringaresinol modulated MAPK- and NF-κB-related signaling in LPS-stimulated macrophages [46]. In contrast, the present study investigated (−)-syringaresinol in TNF-α-stimulated human AGS gastric epithelial cells, demonstrating an association between reduced IL-8 secretion and attenuation of ERK and p38 phosphorylation. The differences in stereochemical form, inflammatory stimulus, cellular context, and biological endpoints distinguish the present findings from previous reports and extend the biological characterization of syringaresinol to gastric epithelial inflammatory responses. However, these differences do not establish stereoselective activity, which would require direct comparison of the two enantiomers under identical experimental conditions.

To further explore potential molecular interactions associated with the observed attenuation of ERK and p38 phosphorylation, molecular docking was performed using MAPK1 (ERK2) and MAPK14 (p38α) as candidate protein targets. Compound **6** exhibited predicted docking scores of −8.117 and −7.206 kcal/mol for MAPK1 and MAPK14, respectively, with several predicted hydrogen-bonding and hydrophobic interactions within the corresponding binding regions. These computational findings provide preliminary structural hypotheses for potential interactions between compound 6 and MAPK proteins but do not establish that such interactions directly account for the observed reductions in ERK and p38 phosphorylation. Indeed, the attenuation of phosphorylation may also arise from modulation of upstream signaling components. Therefore, further direct target-engagement and kinase activity assays are required to determine whether MAPK1 and MAPK14 are direct molecular targets of compound **6** and to clarify their potential contribution to its anti-inflammatory activity.

Collectively, these findings identify syringaldehyde (**2**) and (−)-syringaresinol (**6**) as the most active compounds in the anti-adipogenic and anti-inflammatory screening assays, respectively. The anti-adipogenic activity of compound **2** was associated with attenuation of the PPARγ/C/EBPα-regulated adipogenic differentiation program, whereas the anti-inflammatory activity of compound **6** was accompanied by reduced TNF-α-induced IL-8 production and attenuation of ERK and p38 phosphorylation. These findings extend beyond the initial identification of biological activities by linking the observed cellular effects to changes in molecular pathways involved in adipocyte differentiation and gastric epithelial inflammatory responses. Complementary computational analyses identified *PTGS2* (COX-2) as a predicted common candidate target and suggested potential interactions of compounds **2** and **6** with PPARγ and MAPK proteins, respectively. However, these predictions do not establish direct molecular target engagement, and further biochemical and target-specific functional studies are required to validate the proposed interactions and clarify their contributions to the observed biological activities. Overall, this study expands the phytochemical characterization of *S. glauca* and provides a basis for further investigation of its bioactive constituents and their potential molecular mechanisms.

## 4. Materials and Methods

### 4.1. General Experimental Procedures

Optical rotations were measured using a Jasco P-1020 polarimeter (Jasco, Easton, MD, USA). Ultraviolet (UV) spectra were recorded on an Agilent 8453 UV–visible spectrophotometer (Agilent Technologies, Santa Clara, CA, USA). ^1^H NMR spectra were acquired on a Bruker AVANCE III HD 850 NMR spectrometer (Bruker, Billerica, MA, USA) operating at 850 MHz, with chemical shifts (*δ*) reported in parts per million (ppm) and coupling constants (*J*) in hertz (Hz). Semi-preparative HPLC was performed using a Waters HPLC system equipped with a 2998 photodiode array detector and a 2690 binary pump (Waters Corporation, Milford, CT, USA). LC/MS analysis was conducted on an Agilent 1200 Series HPLC system (Agilent Technologies, Santa Clara, CA, USA) coupled with a diode array detector and a 6130 Series electrospray ionization (ESI) mass spectrometer, using an analytical Kinetex C18 100 Å column (100 mm × 2.1 mm i.d., 5 μm; Phenomenex, Torrance, CA, USA). High-resolution electrospray ionization mass spectra (HR-ESI-MS) were recorded on an Agilent 6545 Q-TOF LC/MS spectrometer (Agilent Technologies, Santa Clara, CA, USA). Thin-layer chromatography (TLC) was carried out on precoated silica gel 60 F_254_ and RP-18 F_254_s plates (Merck, Darmstadt, Germany). Spots were visualized under UV light or by heating after spraying with anisaldehyde–sulfuric acid reagent.

### 4.2. Reagents and Chemicals

Solvents used for extraction and solvent partitioning were of analytical grade and were purchased from Samchun Chemical Co., Ltd. (Seoul, Republic of Korea). Solvents used for chromatographic separation and HPLC purification were of HPLC grade and were purchased from Honeywell International Inc. (Charlotte, NC, USA). All solvents were used as received without further purification. Unless otherwise specified, all commercially available chemicals and reagents were of analytical grade and were used without further purification.

### 4.3. Plant Material

The plant material was collected from an agricultural area adjacent to a waterway in the coastal region of Muan, Jeollanam-do, Republic of Korea (34°59′12.5″ N, 126°25′12.01″ E) in October 2023. This collection site is located in a coastal environment where *S. glauca* naturally grows under saline conditions. However, soil salinity and physicochemical properties were not measured as part of the present study. The plant material was botanically authenticated by Dr. Suk-Kyu Kim of Halopharm Co., Ltd. (Iksan, Republic of Korea). A voucher specimen (NMJ-2023) has been deposited in the herbarium of the School of Pharmacy, Sungkyunkwan University, Suwon, Republic of Korea.

### 4.4. Extraction and Isolation

The dried *Suaeda glauca* (100 g) was ground and extracted with distilled water (2.0 L × 3) under reflux at 95 °C for 1 h. The resulting aqueous extract was filtered under reduced pressure and lyophilized to obtain a powdered extract (5.2 g). The extract was suspended in distilled water (700 mL) and successively partitioned with *n*-hexane, dichloromethane (CH_2_Cl_2_), ethyl acetate (EtOAc), and *n*-butanol (*n*-BuOH) (each 700 mL × 3), affording fractions of 8 mg, 100 mg, 30 mg, and 485 mg, respectively. The CH_2_Cl_2_-soluble fraction (99.8 mg) was subjected to semi-preparative reversed-phase HPLC (Phenomenex C18 100 Å column, 250 × 2.1 mm i.d., 5 μm) using a gradient system of 16–18% acetonitrile (MeCN)/H_2_O, followed by isocratic elution with 25% MeCN/H_2_O at a flow rate of 2 mL/min, to yield compounds **1** (0.6 mg, *t*_R_ = 34.0 min), **2** (0.6 mg, *t*_R_ = 36.0 min), **3** (1.0 mg, *t*_R_ = 48.0 min), **4** (0.6 mg, *t*_R_ = 85.0 min), **5** (1.0 mg, *t*_R_ = 89.0 min), and **6** (1.0 mg, *t*_R_ = 95.0 min).

#### 4.4.1. Vanillin (**1**)

White crystalline solid; UV (MeOH) λ_max_ (log ε) 230 (3.84), 280 (3.76) nm; ESI-MS *m*/*z* 153.1 [M + H]+; HR-ESI-MS (positive ion mode) *m*/*z* 153.0543 [M + H]^+^ (calcd for C_8_H_9_O_3_, 153.0549). The ^1^H NMR data were consistent with those previously reported [33].

#### 4.4.2. Syringaldehyde (**2**)

Pale yellow crystalline solid; UV (MeOH) λ_max_ (log ε) 230 (3.96), 310 (3.94) nm; ESI-MS *m*/*z* 183.0 [M + H]^+^; HR-ESI-MS (positive ion mode) *m*/*z* 183.0648 [M + H]^+^ (calcd for C_9_H_11_O_4_, 183.0654). The ^1^H NMR data were consistent with those previously reported [34].

#### 4.4.3. (–)-Loliolide (**3**)

White crystalline solid; ${\left[\alpha \right]}_{D}^{25}$: −39.0° (*c* 0.01, MeOH), UV (MeOH) λ_max_ (log ε) 225 (2.03) nm; ESI-MS *m*/*z* 197.1 [M + H]^+^; HR-ESI-MS (positive ion mode) *m*/*z* 197.1168 [M + H]^+^ (calcd for C_11_H_17_O_3_, 197.1173). The ^1^H NMR data were consistent with those previously reported [35].

#### 4.4.4. *N*-*trans*-Feruloyltyramine (**4**)

Pale yellow powder; ESI-MS *m*/*z* 314.1 [M + H]^+^; HR-ESI-MS (positive ion mode) *m*/*z* 314.1386 [M + H]^+^ (calcd for C_18_H_20_NO_4_, 314.1387). The ^1^H NMR data were consistent with those previously reported [36].

#### 4.4.5. *N*-*trans*-Feruloylmethoxytyramine (**5**)

Pale yellow powder; ESI-MS *m*/*z* 344.1 [M + H]^+^; HR-ESI-MS (positive ion mode) *m*/*z* 344.1492 [M + H]^+^ (calcd for C_19_H_22_NO_5_, 344.1492). The ^1^H NMR data were consistent with those previously reported [36].

#### 4.4.6. (–)-Syringaresinol (**6**)

Colorless needles; ${\left[\alpha \right]}_{D}^{25}$: −15.6 (*c* 0.03, MeOH); UV (MeOH) λ_max_ (log ε) 240 (2.13) nm; ESI-MS *m*/*z* 441.1 [M + Na]^+^; HR-ESI-MS (positive ion mode) *m*/*z* 441.1516 [M + Na]^+^ (calcd for C_22_H_26_NaO_8_, 441.1519). The ^1^H NMR data were consistent with those previously reported [37].

### 4.5. Cell Culture

The 3T3-L1 mouse preadipocyte cell line and the AGS human gastric adenocarcinoma cell line were purchased from the American Type Culture Collection (ATCC; Manassas, VA, USA). The 3T3-L1 preadipocytes and AGS cells were used to evaluate anti-adipogenic activity and tumor necrosis factor-α (TNF-α)-induced inflammatory responses, respectively. The 3T3-L1 cells were cultured in Dulbecco’s modified Eagle’s medium (DMEM; Corning, NY, USA) supplemented with 10% bovine calf serum (BCS; Gibco, Gaithersburg, MD, USA) and 1% penicillin–streptomycin (Gibco). AGS cells were maintained in RPMI-1640 medium (Corning) supplemented with 10% fetal bovine serum (FBS; Gibco) and 1% penicillin–streptomycin (Gibco). All cells were cultured at 37 °C in a humidified atmosphere containing 5% CO_2_. The culture medium was replaced every 2 days, and the cells were subcultured when they reached approximately 80% confluence. All cell-based experiments were performed in technical triplicate and independently repeated three times.

### 4.6. Cell Viability Assay

Cell viability was assessed using the EZ-Cytox assay. Cells were treated with compounds **1**–**6** at the indicated concentrations for 24 h, followed by incubation with EZ-Cytox reagent according to the manufacturer’s instructions. Absorbance was measured at 450 nm, and viability was expressed as a percentage of the untreated control.

### 4.7. Adipogenic Differentiation of 3T3-L1 Preadipocytes

3T3-L1 preadipocytes were seeded in 48-well plates and cultured in Dulbecco’s Modified Eagle’s Medium (DMEM) supplemented with 10% bovine calf serum (BCS) and 1% penicillin–streptomycin (P/S). After reaching confluence, the cells were maintained for an additional 2 days in DMEM containing 10% fetal bovine serum (FBS) and 1% P/S. Adipogenic differentiation was induced as previously described [47] using differentiation medium containing 10% FBS, insulin, dexamethasone, and 3-isobutyl-1-methylxanthine (IBMX), in the presence or absence of the test compounds or atorvastatin as a positive control. After 72 h, the medium was replaced with maintenance medium containing insulin and the corresponding test compounds or positive control. Thereafter, the medium was refreshed every 2 days until the completion of differentiation.

### 4.8. Western Blot Analysis of Adipogenic Proteins

3T3-L1 cells were differentiated in 6-well plates in the presence of compound **2** or atorvastatin (positive control) at the indicated concentrations [48,49,50]. Following differentiation, the cells were washed with phosphate-buffered saline (PBS) and lysed in RIPA buffer supplemented with protease and phosphatase inhibitor cocktails. Protein concentrations were determined using a BCA protein assay kit. Equal amounts of protein were separated by SDS-polyacrylamide gel electrophoresis (SDS-PAGE) and transferred onto polyvinylidene difluoride (PVDF) membranes. The membranes were blocked and incubated with primary antibodies against peroxisome proliferator-activated receptor gamma (PPARγ), CCAAT/enhancer-binding protein alpha (C/EBPα), adiponectin, and β-actin (Cell Signaling Technology, Danvers, MA, USA), followed by incubation with the corresponding horseradish peroxidase-conjugated secondary antibodies. Protein bands were visualized using an enhanced chemiluminescence (ECL) detection system and quantified by densitometric analysis. The expression levels of target proteins were normalized to β-actin.

### 4.9. Measurement of TNF-α-Induced IL-8 Secretion

AGS cells were pretreated with compounds **1**–**6** at concentrations ranging from 6.25 to 200 μM, followed by stimulation with TNF-α (20 ng/mL) to induce an inflammatory response, as previously described [6,43]. N-Acetyl-L-cysteine (NAC, 3200 μg/mL) was used as a positive control. After incubation, culture supernatants were collected, and IL-8 levels were quantified using a Human IL-8 ELISA Kit (R&D Systems, Minneapolis, MN, USA) according to the manufacturer’s instructions.

### 4.10. Western Blot Analysis of MAPK and AKT Signaling

AGS cells were pretreated with compound **6** (100 or 200 μM) or N-acetyl-L-cysteine (NAC, 3200 μg/mL) and subsequently stimulated with TNF-α (20 ng/mL). Following treatment, cells were lysed in RIPA buffer supplemented with protease and phosphatase inhibitor cocktails. Protein concentrations were determined using a BCA protein assay kit. Equal amounts of protein were separated by SDS-polyacrylamide gel electrophoresis (SDS-PAGE) and transferred onto polyvinylidene difluoride (PVDF) membranes. The membranes were incubated with primary antibodies against phosphorylated ERK (p-ERK), ERK, phosphorylated p38 (p-p38), p38, phosphorylated JNK (p-JNK), JNK, phosphorylated AKT (p-AKT), AKT, and β-actin (Cell Signaling Technology, Danvers, MA, USA), followed by incubation with the corresponding horseradish peroxidase-conjugated secondary antibodies. Protein bands were visualized using an enhanced chemiluminescence (ECL) detection system and quantified by densitometric analysis. The levels of phosphorylated ERK, p38, JNK, and AKT were normalized to their corresponding total protein levels, while β-actin was used as a loading control [51,52].

### 4.11. Statistical Analysis

All data are presented as the mean ± standard deviation (SD) from three independent experiments. Statistical analyses were performed using one-way analysis of variance (ANOVA) followed by Tukey’s multiple comparison test. Differences were considered statistically significant at *p* < 0.05. Statistical significance is indicated as follows: * *p* < 0.05, ** *p* < 0.01, and *** *p* < 0.001.

### 4.12. Target Prediction and Disease Network Analysis

Potential macromolecular targets for compound **2** (syringaldehyde) and compound **6** ((–)-syringaresinol) were predicted utilizing the SwissTargetPrediction web server (https://www.swisstargetprediction.ch/, accessed on 3 September 2026) [53]. To establish the clinical relevance of these targets, disease-associated gene networks for obesity and gastric inflammation were retrieved from the DisGeNET database (https://disgenet.com/, accessed on 3 September 2026). The intersection of the chemical compound-predicted targets and the DisGeNET disease datasets was critically analyzed to identify shared therapeutic genes bridging the metabolic and inflammatory pathways.

### 4.13. Protein and Ligand Preparation

Crystal structures for the protein targets—MAPK1 (PDB ID: 5K4I), MAPK14 (PDB ID: 9MHB), and PPAR-γ (PDB ID: 5GTP)—were retrieved from the RSCB Protein Data Bank (https://www.rcsb.org/, accessed on 3 September 2026). Structural gaps, including the unresolved activation loops in the MAPK structures, were repaired via UCSF ChimeraX [54] and Modeller [55] using the dope-hr (High-Resolution Discrete Optimized Protein Energy) protocol. UniProt biological sequences were aligned and missing internal structures were modeled using two adjacent flexible residues. The model exhibiting the most negative zDOPE score was selected for further studies. Receptor structures were minimized and prepared using OpenBabel, which added physiological hydrogens (pH 7.4), computed Gasteiger charges, merged non-polar hydrogens, and exported rigid PDBQT files. Ligands (SDF format) were parameterized using the Meeko Python module (mk_prepare_ligand.py) to generate Vina-compatible PDBQT files with dynamic torsion angles.

### 4.14. Molecular Docking Protocol

Molecular docking was performed using AutoDock Vina (AutoDock Vina 7ac2999-mod) [56]. The search space was defined by a specific 3D grid box (e.g., center: X = −18.09, Y = 4.523, Z = −18.338; dimensions: 50.024 × 50.024 × 60.606 Å) encompassing the functional binding pockets. The docking algorithm utilized a rigorous exhaustiveness setting of 124, generating a maximum of 50 binding modes with a 3.0 kcal/mol energy range threshold to ensure deep conformational sampling.

## 5. Conclusions

Phytochemical investigation of *S. glauca* resulted in the isolation of six compounds (**1**–**6**), four of which (**1**–**3** and **6**) are reported from this species for the first time. Among the isolated compounds, syringaldehyde (**2**) exhibited anti-adipogenic activity in 3T3-L1 cells, accompanied by reduced lipid accumulation and decreased expression of PPARγ, C/EBPα, and adiponectin, suggesting suppression of adipocyte differentiation and maturation. In addition, (−)-syringaresinol (**6**) attenuated TNF-α-induced inflammatory responses in AGS cells, as evidenced by reduced IL-8 secretion and decreased phosphorylation of ERK and p38 MAPKs. These findings expand the phytochemical knowledge of *S. glauca* and provide experimental evidence for the anti-adipogenic and anti-inflammatory activities of its isolated constituents. Nevertheless, the present findings are limited to in vitro cellular models, and further studies, including direct molecular target validation and in vivo investigations, are required to clarify the underlying mechanisms and evaluate the broader biological potential of these compounds.

## Figures and Tables

**Table 1:** Molecular Docking and Binding Affinity for compounds **2** and **6**.

Compound	Target Protein	Docking Score (kcal/mol)
Compound **2**	PPAR-γ (PDB ID: 5GTP)	−5.306
Compound **6**	MAPK1 (PDB ID: 5K4I)	−8.117
	MAPK14 (PDB ID:9MHB)	−7.206

## Data Availability

The data supporting this study’s findings are available from the corresponding authors upon reasonable request.

## Supplementary Materials

The following supporting information can be downloaded at: https://www.mdpi.com/article/10.3390/md24100368/s1, File S1: Genomic information from DisGeNET and SwissTargetPrediction Databases; Figure S1: The ^1^H NMR spectrum of **1** (CD_3_OD, 850 MHz); Figure S2: The HR-ESIMS (positive ion mode) data of **1**; Figure S3: The ^1^H NMR spectrum of **2** (CD_3_OD, 850 MHz); Figure S4: The HR-ESIMS (positive ion mode) data of **2**; Figure S5: The ^1^H NMR spectrum of **3** (CD_3_OD, 850 MHz); Figure S6: The HR-ESIMS (positive ion mode) data of **3**; Figure S7: The ^1^H NMR spectrum of **4** (CD_3_OD, 850 MHz); Figure S8: The HR-ESIMS (positive ion mode) data of **4**; Figure S9: The ^1^H NMR spectrum of **5** (CD_3_OD, 850 MHz); Figure S10: The HR-ESIMS (positive ion mode) data of **5**; Figure S11: The ^1^H NMR spectrum of **6** (CD_3_OD, 850 MHz); Figure S12: The HR-ESIMS (positive ion mode) data of **6**.
