# Supplementary material for: Chemical Constituents of Halophyte Suaeda glauca and Their Anti-Adipogenic and Anti-Inflammatory Activities
Source: Mar Drugs. 2026 Oct 20;24(10):368. doi: 10.3390/md24100368 (PMC13707108; doi:10.3390/md24100368)

Supplementary Materials

Chemical Constituents of Halophyte *Suaeda glauca* and Their Anti-Adipogenic and Anti-Inflammatory Activities

Supplementary Materials

Figure S1. The ^{1}H NMR spectrum of **1** (CD_3OD, 850 MHz) S3

Figure S2. The HR-ESIMS (positive ion mode) data of **1** S4

Figure S3. The ^{1}H NMR spectrum of **2** (CD_3OD, 850 MHz) S5

Figure S4. The HR-ESIMS (positive ion mode) data of **2** S6

Figure S5. The ^{1}H NMR spectrum of **3** (CD_3OD, 850 MHz) S7

Figure S6. The HR-ESIMS (positive ion mode) data of **3** S8

Figure S7. The ^{1}H NMR spectrum of **4** (CD_3OD, 850 MHz) S9

Figure S8. The HR-ESIMS (positive ion mode) data of **4** S10

Figure S9. The ^{1}H NMR spectrum of **5** (CD_3OD, 850 MHz) S11

Figure S10. The HR-ESIMS (positive ion mode) data of **5** S12

Figure S11. The ^{1}H NMR spectrum of **6** (CD_3OD, 850 MHz) S13

Figure S12. The HR-ESIMS (positive ion mode) data of **6** S14

Figure S1. The ^{1}H NMR spectrum of **1** (CD_3OD, 850 MHz)

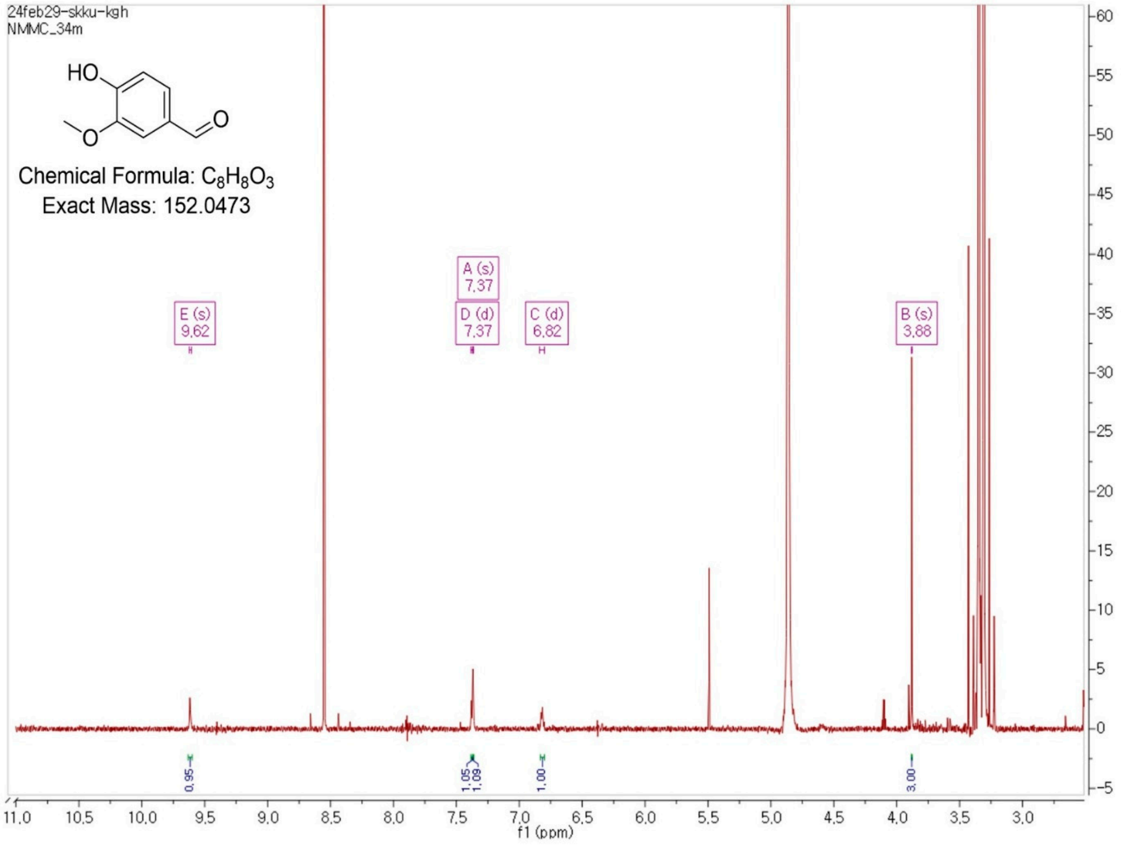

^{1}H NMR (CD_3OD, 850 MHz): δ 9.62 (1H, s, CHO), 7.37 (1H, d, J = 7.5 Hz, H-6), 7.37 (1H, s, H-2), 6.82 (1H, d, J = 7.5 Hz, H-5), 3.88 (3H, s, -OCH_3).

Figure S2. The HR-ESIMS (positive ion mode) data of **1**

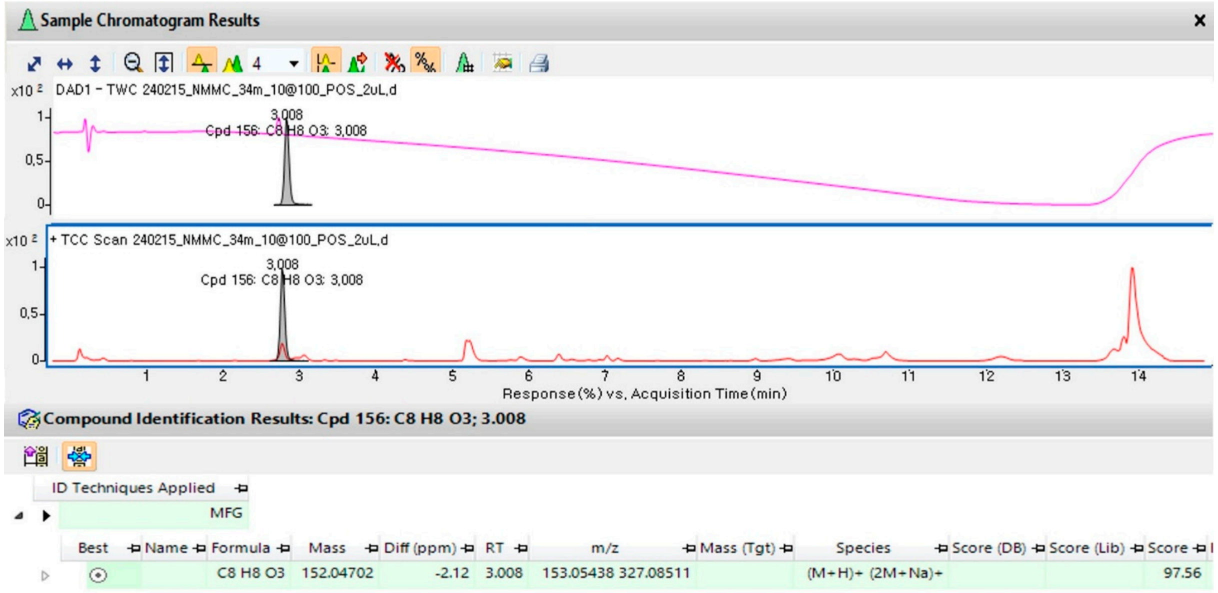

Figure S3. The ^{1}H NMR spectrum of **2** (CD_3OD, 850 MHz)

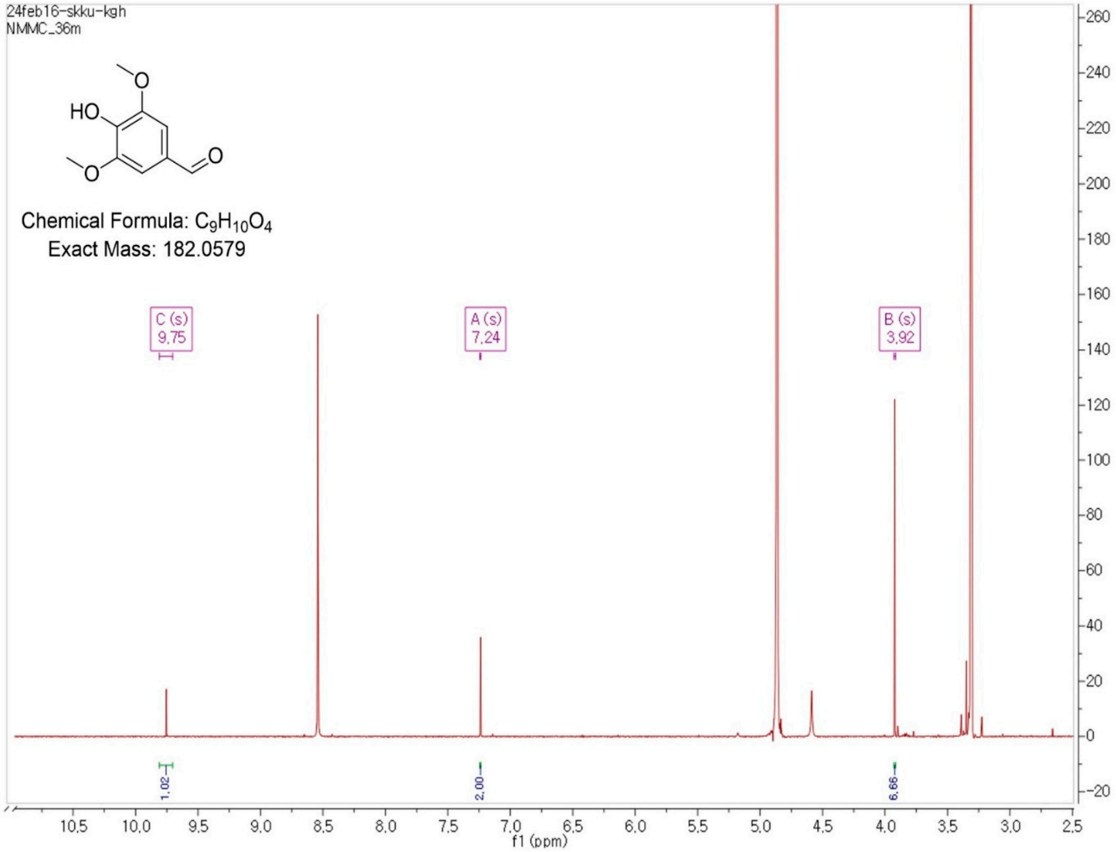

^{1}H NMR (CD_3OD, 850 MHz): δ 9.75 (1H, s, CHO), 7.24 (2H, s, H-2,6), 3.92 (6H, s, 3-OCH_3 and 5-OCH_3).

Figure S4. The HR-ESIMS (positive ion mode) data of **2**

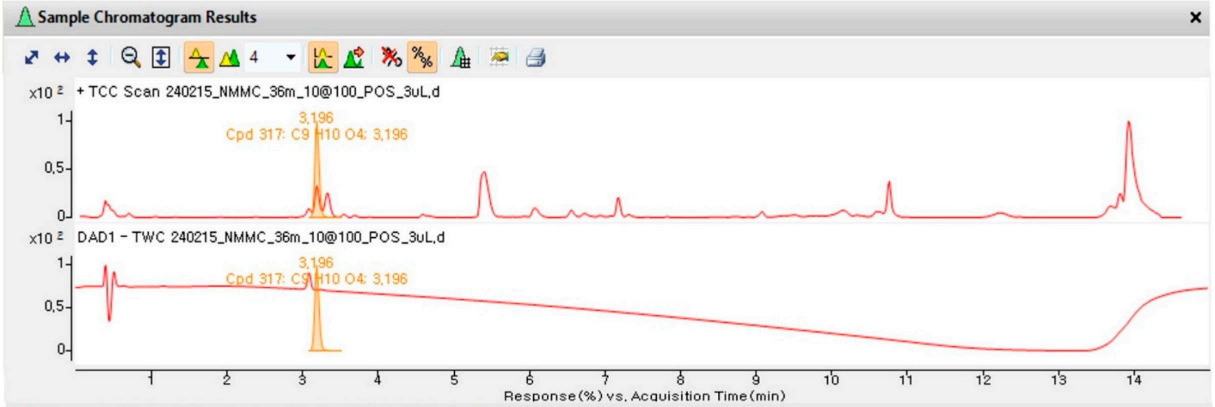

Compound Identification Results: Cpd 317: C9 H10 O4; 3.196

ID Techniques Applied: MFG

Score (Tg	Best	Name	Formula	Mass	Diff (ppm)	RT	m/z	Mass	Species	Score	Score	Flags
	⊙		C9 H10 O4	182.05758	-1.82	3.196	183.06487 205.04592		(M+H)+ (M+Na)+		86.75	

Figure S5. The ^{1}H NMR spectrum of **3** (CD_3OD, 850 MHz)

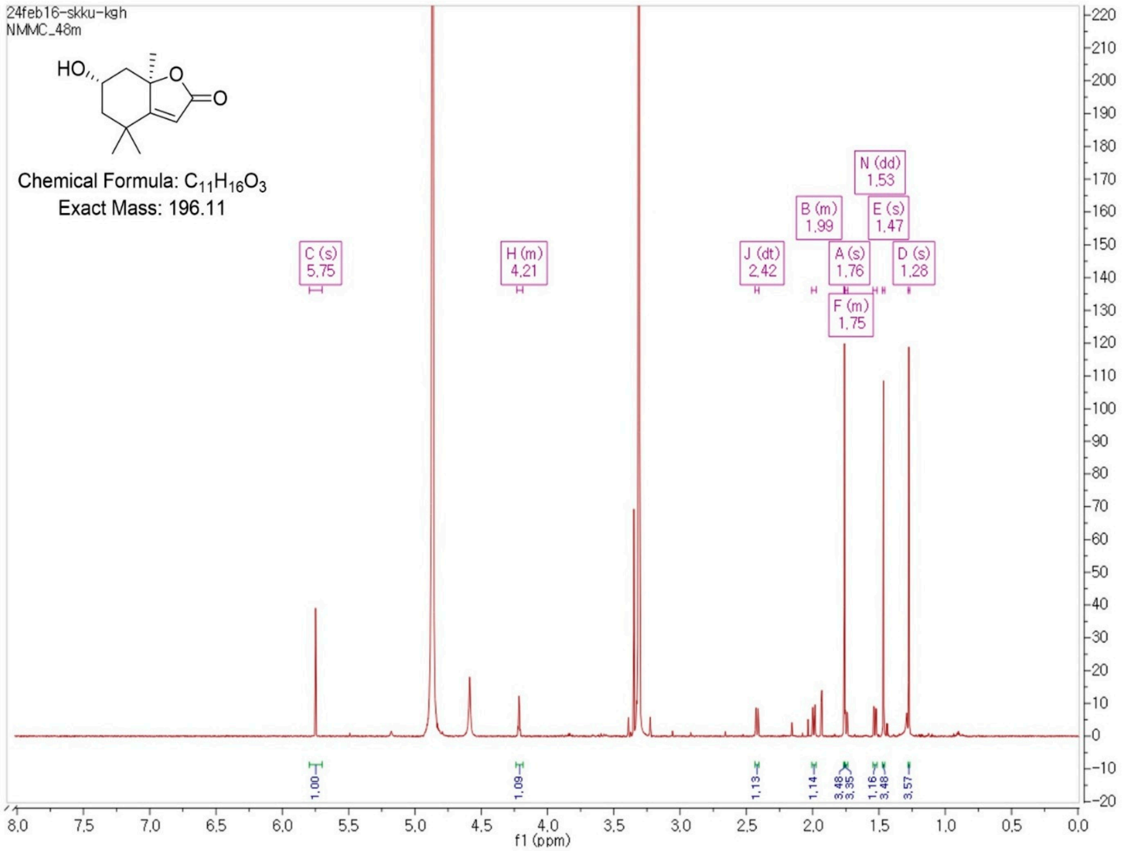

^{1}H NMR (CD_3OD, 850 MHz): δ 5.75 (1H, s, H-3), 4.21 (1H, m, H-6), 2.42 (1H, dt, J = 13.5, 2.5 Hz, H-7a), 1.99 (1H, m, H-5a), 1.76 (3H, s, H-10), 1.75 (1H, m, H-7b), 1.53 (1H, dd, J = 14.5, 3.5 Hz, H-5b), 1.47 (3H, s, H-8), 1.28 (3H, s, H-9).

Figure S6. The HR-ESIMS (positive ion mode) data of **3**

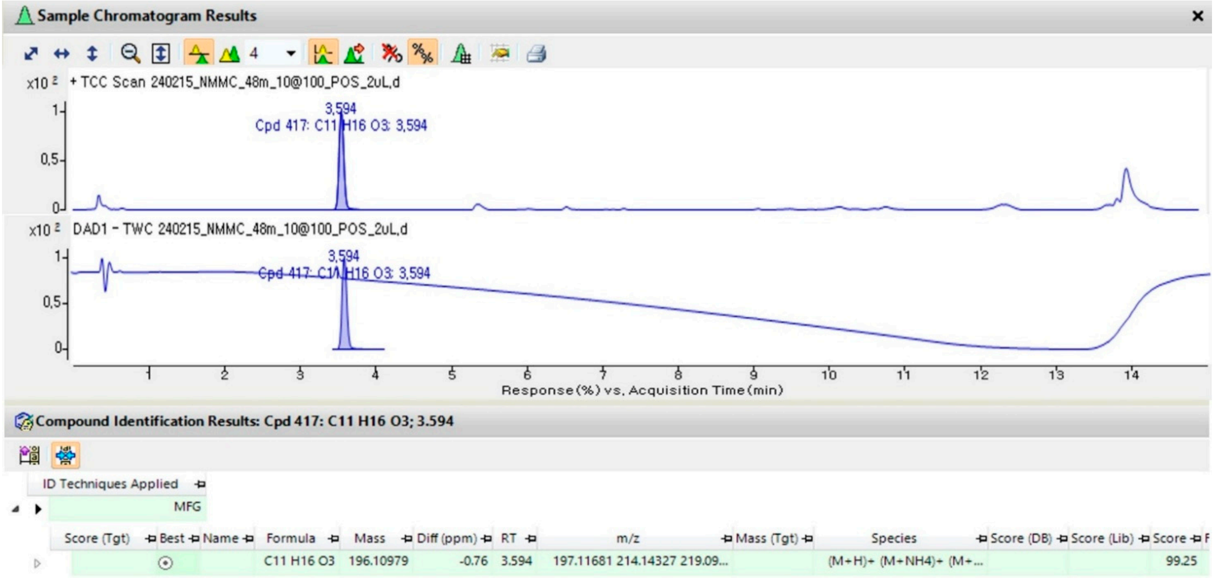

Figure S7. The ¹H NMR spectrum of **4** (CD_3OD, 850 MHz)

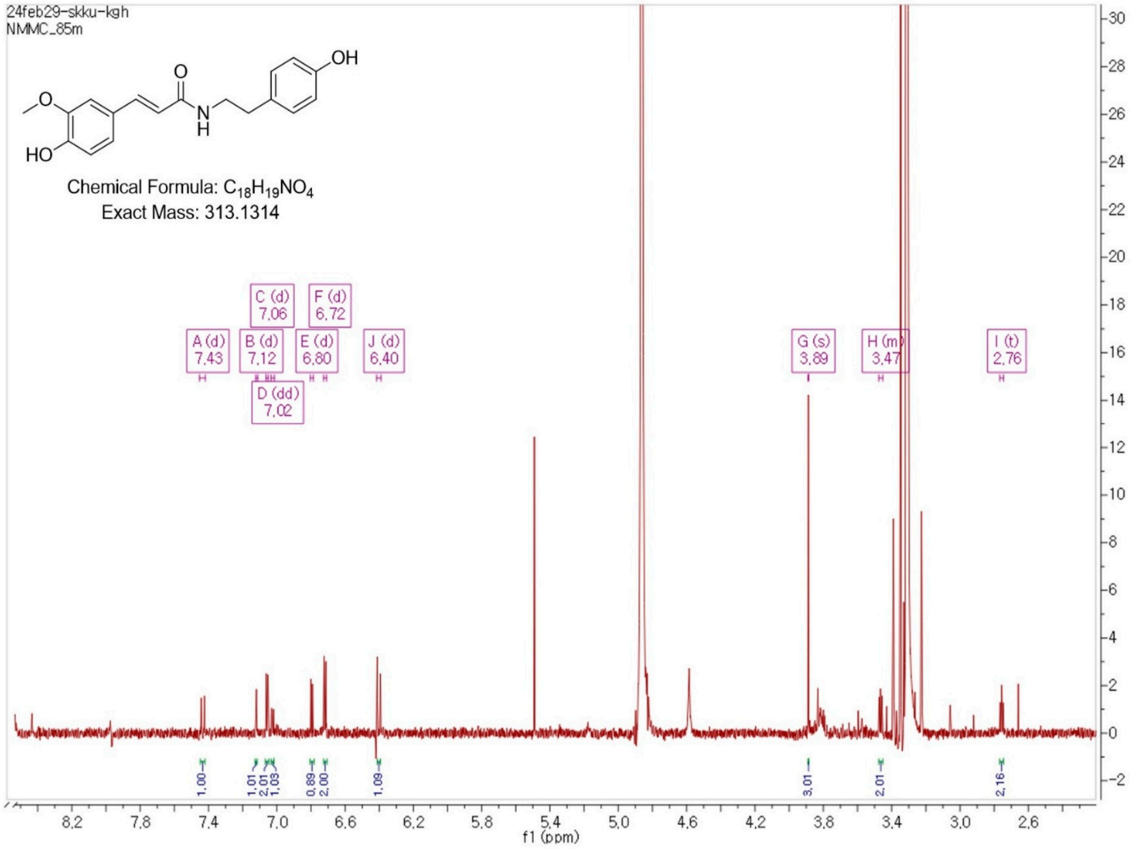

¹H NMR (CD_3OD, 850 MHz): δ 7.43 (1H, d, J = 16.0 Hz, H-7), 7.12 (1H, d, J = 2.0 Hz, H-2), 7.06 (2H, d, J = 8.5 Hz, H-2′,6′), 7.02 (1H, dd, J = 8.0, 2.0 Hz, H-6), 6.80 (1H, d, J = 8.0 Hz, H-2), 6.72 (2H, d, J = 8.5 Hz, H-3′,5′), 6.40 (1H, d, J = 16.0 Hz, H-8), 3.89 (3H, s, -OCH_3), 3.47 (2H, m, H-8′), 2.76 (2H, t, J = 7.5 Hz, H-7′).

Figure S8. The HR-ESIMS (positive ion mode) data of **4**

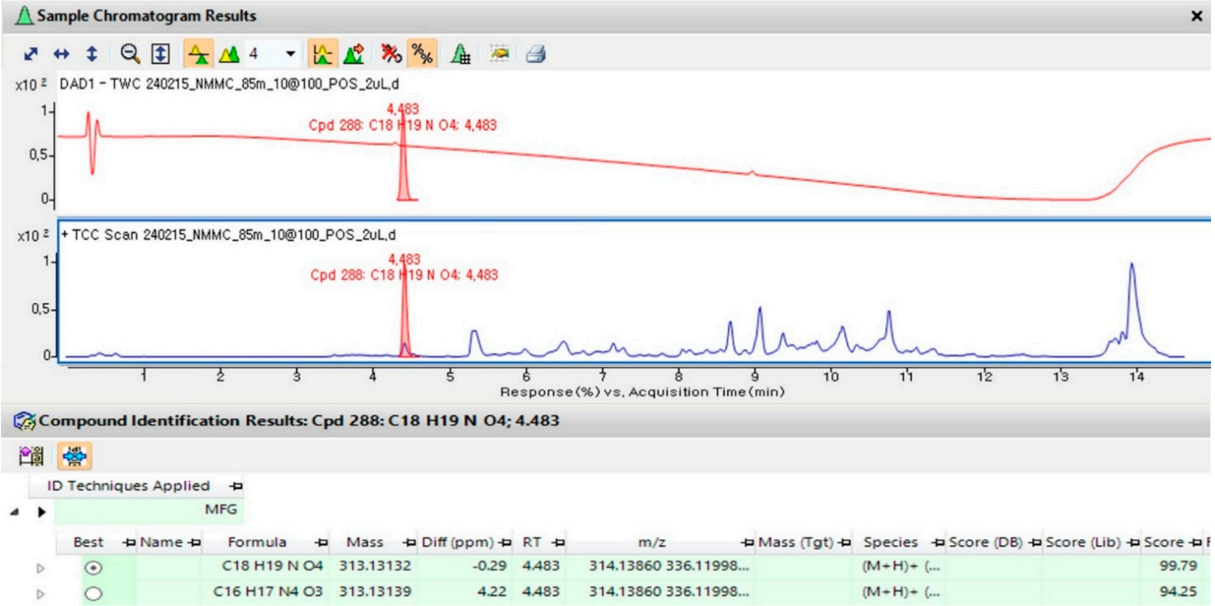

Figure S9. The ^{1}H NMR spectrum of **5** (CD_3OD, 850 MHz)

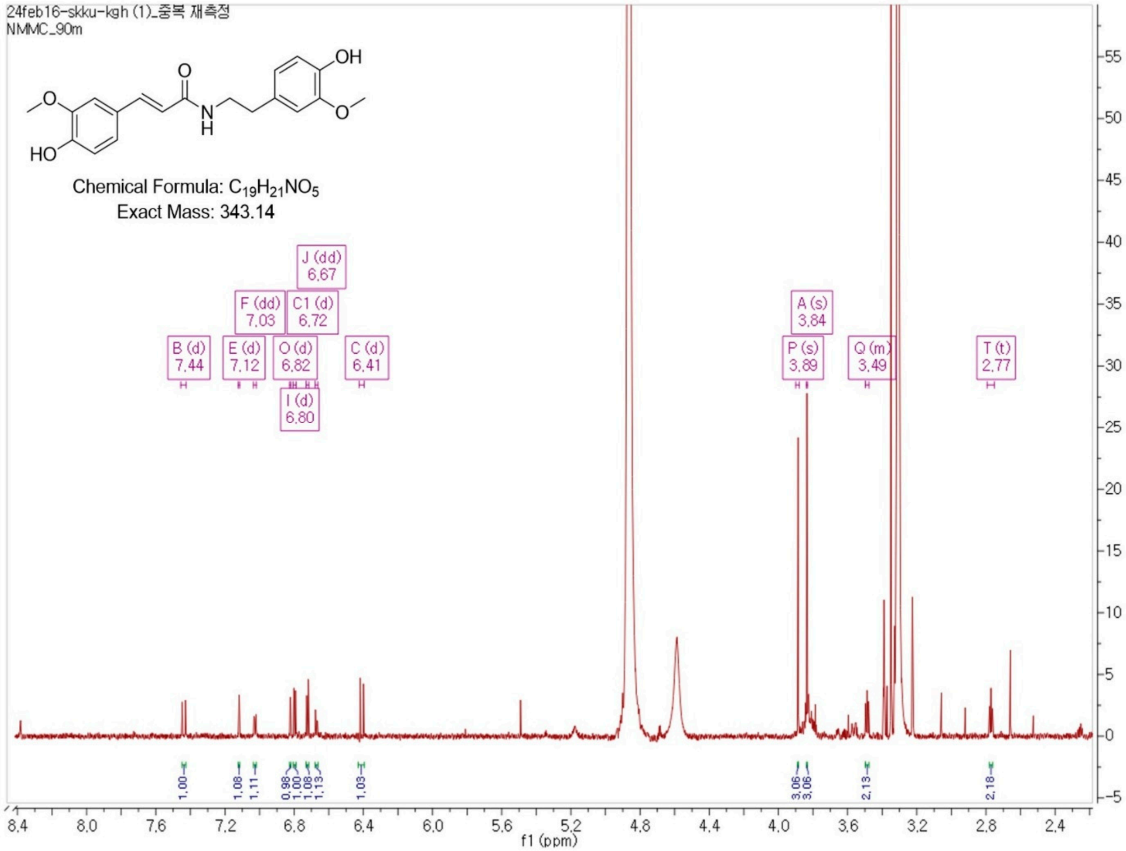

^{1}H NMR (CD_3OD, 850 MHz): δ 7.43 (1H, d, J = 16.0 Hz, H-7), 7.12 (1H, d, J = 2.0 Hz, H-2), 7.06 (2H, d, J = 8.5 Hz, H-2′,6′), 7.02 (1H, dd, J = 8.0, 2.0 Hz, H-6), 6.80 (1H, d, J = 8.0 Hz, H-2), 6.72 (2H, d, J = 8.5 Hz, H-3′,5′), 6.40 (1H, d, J = 16.0 Hz, H-8), 3.89 (3H, s, $-OCH_3$), 3.47 (2H, m, H-8′), 2.76 (2H, t, J = 7.5 Hz, H-7′).

Figure S10. The HR-ESIMS (positive ion mode) data of **5**

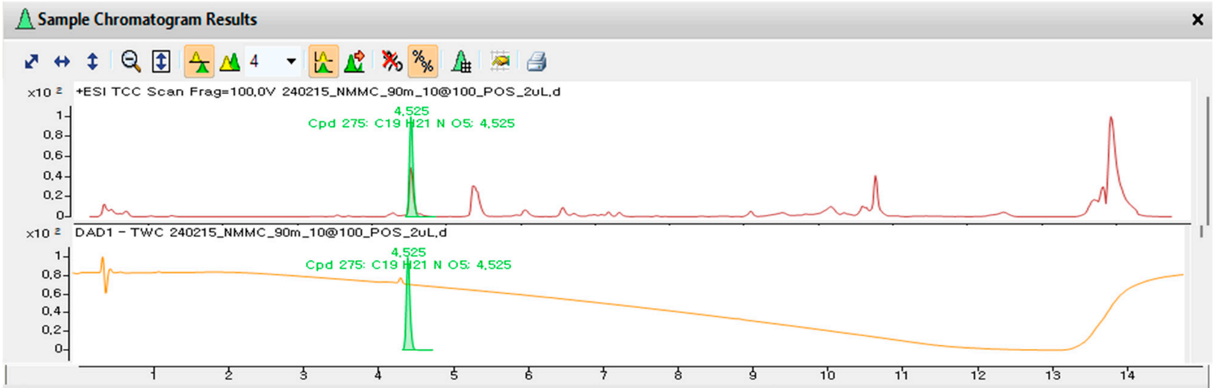

Compound Identification Results: Cpd 275: C19 H21 N O5; 4.525

ID Techniques Applied
MFG

Best	Name	Formula	Mass	Diff (ppm)	RT	m/z	Species	Score	Mass (Tgt)	Score (Tgt)
⦿		C19 H21 N O5	343.1416	-1.01	4.525	344.1492 366.1309 687.2906 709.2706	(M+H)+ (M+Na)+ (2M+H)+ (2M+Na)+	99.37		
○		C16 H23 O8	343.1416	6.77	4.525	344.1492 366.1309 687.2906 709.2706	(M+H)+ (M+Na)+ (2M+H)+ (2M+Na)+	85.12		

Figure S11. The ^{1}H NMR spectrum of **6** (CD_3OD, 850 MHz)

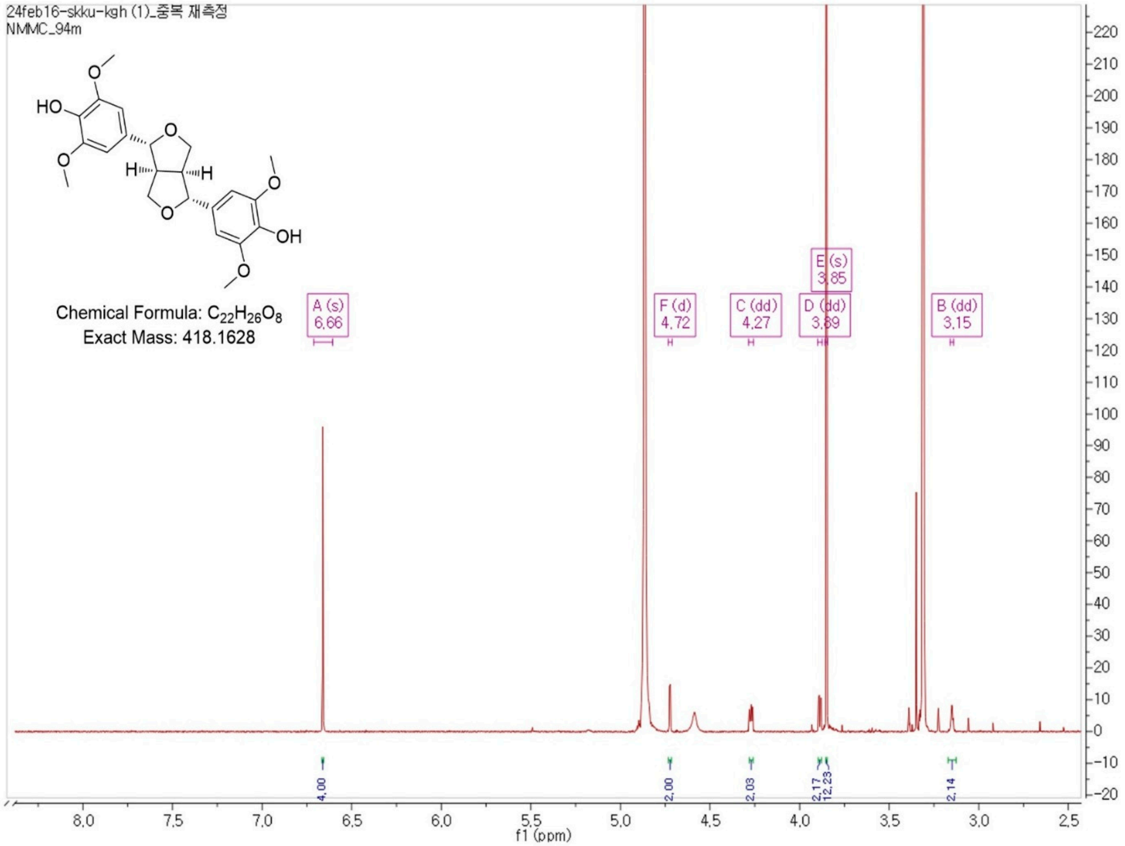

^{1}H NMR (CD_3OD, 850 MHz): δ 6.66 (4H, s, H-2,2′,6,6′), 4.72 (2H, d, J = 4.5 Hz, H-7,7′), 4.27 (2H, dd, J = 9.0, 7.0 Hz, H-9b,9′b), 3.89 (2H, dd, J = 9.0, 3.5 Hz, H-9a,9′a), 3.85 (12H, s, 3,3′,5,5′-OCH_3), 3.15 (2H, dd, J = 6.5, 5.0 Hz, H-8,8′).

Figure S12. The HR-ESIMS (positive ion mode) data of **6**

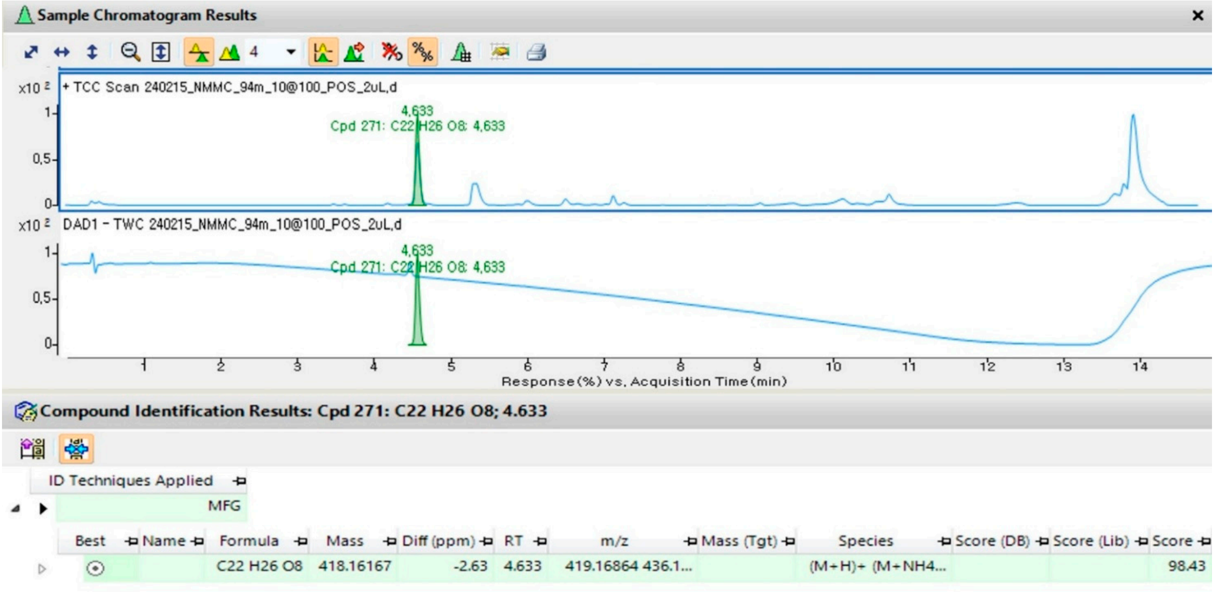

Supplement: Supplementary file 1 [file marinedrugs-24-00368-s001.zip › Supplementary Figures.pdf]
